# Three-Dimensional Point Cloud Applications, Datasets, and Compression Methodologies for Remote Sensing: A Meta-Survey

**DOI:** 10.3390/s25061660

**Published:** 2025-03-07

**Authors:** Emil Dumic, Luís A. da Silva Cruz

**Affiliations:** 1 Department of Electrical Engineering, University North, 104. Brigade 3, 42000 Varaždin, Croatia; 2 Department of Electrical and Computer Engineering, University of Coimbra, 3030-290 Coimbra, Portugal; lcruz@deec.uc.pt; 3 Instituto de Telecomunicações, 3030-290 Coimbra, Portugal

**Keywords:** point cloud, remote sensing, point cloud datasets, point cloud compression

## Abstract

This meta-survey provides a comprehensive review of 3D point cloud (PC) applications in remote sensing (RS), essential datasets available for research and development purposes, and state-of-the-art point cloud compression methods. It offers a comprehensive exploration of the diverse applications of point clouds in remote sensing, including specialized tasks within the field, precision agriculture-focused applications, and broader general uses. Furthermore, datasets that are commonly used in remote-sensing-related research and development tasks are surveyed, including urban, outdoor, and indoor environment datasets; vehicle-related datasets; object datasets; agriculture-related datasets; and other more specialized datasets. Due to their importance in practical applications, this article also surveys point cloud compression technologies from widely used tree- and projection-based methods to more recent deep learning (DL)-based technologies. This study synthesizes insights from previous reviews and original research to identify emerging trends, challenges, and opportunities, serving as a valuable resource for advancing the use of point clouds in remote sensing.

## 1. Introduction

Three-dimensional point clouds (PCs) have always attracted substantial attention in remote sensing (RS) because of their ability to accurately represent complex 3D structures and surfaces. Typically, point clouds are represented as a set of distinct points in a three-dimensional space (so-called geometric information), possibly with one or more attribute components per point, such as color, reflectance, temperature, or other variables of interest. PC data are often obtained using advanced sensing technologies such as light detection and ranging (LiDAR), photogrammetry, radio detection and ranging (RADAR), synthetic aperture RADAR (SAR), sound detection and ranging (SONAR), and other 3D scanning methods. These types of data are very important and widely used in remote sensing applications such as environmental monitoring, urban planning, forestry, and disaster management, as they provide precise spatial information regarding an object’s location and dimensions, as well as terrain topography.

The need for solving problems related to the efficient storage, transmission, and processing of such massive datasets has increased, along with the usage of point cloud data in remote sensing. Point clouds, which are sometimes highly detailed, tend to involve enormous amounts of data, which makes their handling and use challenging. To address this problem, efficient point cloud data compression has emerged as an important research area, and various methodologies have been proposed to reduce point cloud data size while preserving geometric accuracy and attribute representation fidelity. Newer compression methodologies based on deep learning (DL) are of special interest as they are a fast-evolving alternative to earlier non-DL compression approaches.

This article is organized into three main sections, describing different aspects of PC applications in RS:

- Section 2: A meta-survey of RS-related PC applications;
- Section 3: PC datasets for RS-related tasks;
- Section 4: PC compression methodologies.

Section 2 provides a review of articles that describes the different PC applications in RS explored in this study. The surveyed articles are organized into three subsections: general PC-related, specific RS-related, and agriculture-related applications. Next, Section 3 introduces several PC datasets used in research and algorithmic development, classified into six categories: urban scenes, outdoor- and vehicle-related contexts, indoor scenarios, small-size and medium-size object representation, agriculture-related contexts, and other application-specific datasets. Section 4 covers different PC compression methods, and they are divided into several categories: common tree-based point cloud compression, projection-based point cloud compression; voxelized or octree-based static or dynamic PC geometry compression; point-based point cloud compression; attribute compression; emerging neural radiance field (NeRF)-based PC compression; and other point cloud compression methods and point cloud compression applications. Finally, Section 5 presents conclusions and future research.

## 2. Point Cloud Applications in Remote Sensing

The following paragraphs present a review of survey articles that describe the applications of PCs in RS activities. A total of 59 survey articles published in several journals in the past 10 years were selected for review using the Scopus database, with the keywords “point cloud” and “remote sensing” applied. Some articles that are not surveys but present original research results were included due to their importance and relevance to this meta-survey.

**Figure 1:**
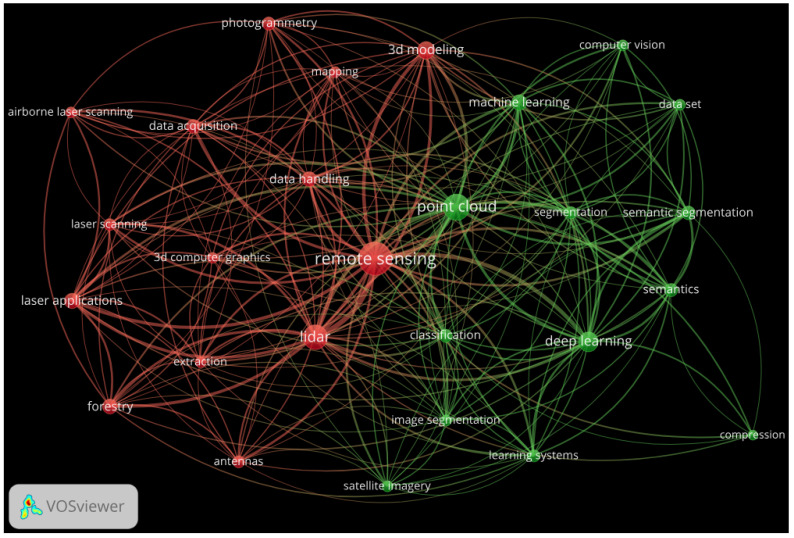
VOSviewer results, using at least 5 overlapping keywords (except compression keyword) from 59 review articles described earlier in the section, showing a total of 27 keywords. Line width represents the normalized strength of the link between two keywords, i.e., the number of joint keywords from analyzed papers. Circle size represents the weight of the specific keyword, i.e., the number of occurrences in analyzed papers. Different colors are used to represent keyword clustering (two clusters in this case). For more information, consult the observations in [1].

We also present some information about keyword occurrences and the co-occurrence frequency of the surveyed papers. We used VOSviewer [1] to prepare the keyword occurrence graph shown in Figure 1. We used at least five occurrences (common keywords) from all reviews, except for the “compression” keyword (with four occurrences); combined words with similar meaning; removed the word “review”; and graphically represented the relationships between the 27 selected keywords. Figure 1 illustrates the topics frequently analyzed alongside the searched keywords “point cloud” and “remote sensing”. This visualization highlights the relationships between various themes and concepts, listing the areas of research and application commonly associated with these keywords.

It can be concluded that the composite keyword "point cloud" belongs to the same (green) cluster as the 12 keywords: classification, compression, computer vision, dataset, deep learning, image segmentation, learning systems, machine learning, satellite imagery, segmentation, semantic segmentation, and semantics. Similarly, the composite keyword “remote sensing”, which belongs to the red cluster, is associated with 13 keywords: 3D computer graphics, 3D modeling, airborne laser scanner, antennas, data acquisition, data handling, extraction, forestry, laser applications, laser scanning, LiDAR, mapping, and photogrammetry.

The keyword “dataset”, with five occurrences, discussed in Section 3, is linked to multiple terms across both clusters. In the green cluster, it connects to computer vision, deep learning, and machine learning, among others, while in the red cluster, it is associated with 3D modeling, LiDAR, and remote sensing.

The keyword “compression”, with four occurrences, discussed in Section 4, is associated with five keywords from the green cluster—classification, deep learning, point cloud, semantics, and semantic segmentation—and two keywords from the red cluster: remote sensing and LiDAR. It can be observed that compression analysis has received relatively limited attention in review articles focusing on PCs in RS. Therefore, PC compression algorithms will also be reviewed in depth in Section 4.

**Figure 2:**
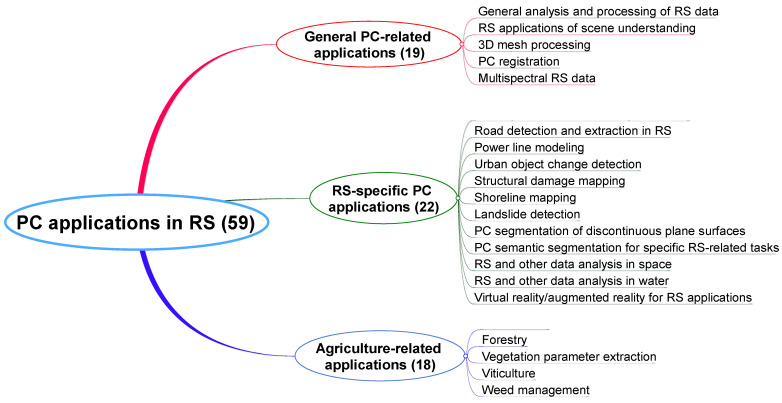
Map of discussed point cloud applications in remote sensing.

The following subsections discuss several applications, categorized into three groups according to the selected review articles, as shown in Figure 2: general PC-related, specific RS-related, and agriculture-related applications.

Additional research discussing other aspects of the capture and use of PC are reported by the authors of [2], who address data acquisition technologies, intelligent processing algorithms, and their applications in RS in scientific and engineering contexts. Newer collections of research also include two editorials, with the authors of [3] focusing on intelligent PC processing, sensing, and understanding and the authors of [4] exploring PC processing with machine learning techniques. A noteworthy source of information is provided by the authors of [5], who introduce advanced theories and methodologies for AI-driven PC processing, with applications to earth observation, 3D vision, autonomous driving, smart cities, and geospatial information systems.

### 2.1. General Point Cloud-Related Applications

This subsection summarizes different review papers focused on the general applications of PC to RS tasks. The studies surveyed are listed in Table 1, which provides information about the year of publication, type of platform used in the work (ground-based, aerial, etc.) and type of application.

#### 2.1.1. General Analysis and Processing of Remote Sensing Data

The article by Camuffo et al. [6] reviews recent DL-based PC processing algorithms for semantic scene understanding (classification, detection, and semantic segmentation (SS)), compression, and PC completion. In contrast to previous, less structured studies, this study suggests a new taxonomical classification of the methods covered based on variables such as the setup for the acquisition, the properties of the PC data that are acquired, data formatting, side information inclusion, and the features of DL architectures. This classification identifies areas for future research and presents performance evaluations using well-established datasets. The compression models discussed in this study, along with several others, are explained in detail in Section 4.

The authors of [7] examine different applications of computer vision and pattern recognition methods relative to RS data for change detection, boundary extraction, land cover mapping, and target detection. They include a variety of imaging modalities such as digital elevation models (DEMs); LiDAR PCs; and multispectral, hyperspectral, and SAR imagery.

In [2], Yang et al. address current research directions and trends in three areas: point cloud big data acquisition (scanner types and scanner platforms); PC processing (such as denoising, completion, registration, segmentation, and surface reconstruction); and different engineering applications (such as geospatial information, smart cities, underground space development, infrastructure construction, automotive industry, and cultural heritage).

The authors of [8] provide an overview of current techniques for compressing point cloud data from vehicular LiDAR sensors. They present a comprehensive classification that categorizes these methods into four main groups: coding-based, format-based, 2D, and 3D compression. The article evaluates these methods based on key performance metrics such as the compression ratio (CR), bits per point (bpp), and point-to-plane error measures such as the MSE and PSNR.

In [9], Martins et al. characterize the impact of LiDAR PC compression on object detection evaluated in the Kitti dataset [24]. The article presents a thorough review of LiDAR PC compression methods (including learning-based methods) and object detection methods. The compression models used were modified JPEG Pleno PC coding [25], G-PCC [26], and L3C2 [27], and they are described in more detail in Section 4. The authors indicate the availability of the datasets prepared for the study, which can be provided upon request.

#### 2.1.2. Remote Sensing Applications of Scene Understanding

State-of-the-art approaches to mobile laser scanner (MLS) data processing are summarized by the authors of [10]. The tasks reviewed include segmentation based on feature extraction, object detection, SS, and feature extraction (i.e., low-level properties such as edge detection). The benchmark datasets that are currently available for SS and object recognition are also listed.

Point cloud semantic segmentation (PCSS), which extends SS in 2D images to 3D images by employing irregularly dispersed points in 3D space rather than regularly distributed pixels in 2D images, is described by the authors of [11]. Point clouds can be produced from stereo or multiview imagery, or they can be directly acquired via distance-measuring devices. Progress in stereovision algorithms and diverse 3D sensors has enabled the easy generation of 3D point clouds.

Yuan et al., the authors of [12], reviewed recent developments in DL and basic deep neural network designs to perform SS in RS data, including novel data types such as PCs and hyperspectral images. When compared to applications in satellite imaging, recent techniques typically perform poorly on unconventional, unstructured PCs and rich spectral images. According to the authors, learning from very small datasets results in a performance gap, showing that the limited availability of labeled non-conventional RS data presents a major challenge to the development and assessment of novel deep learning techniques.

In [13], two distinct aerial LiDAR datasets are used to conduct a thorough evaluation of three popular DL networks for PCSS: PointNet++, SparseCNN, and KPConv. These networks are assessed for generalization, computation time, classification accuracy, and sensitivity to changes in hyper-parameters.

The authors of [14] review advanced DL models for LiDAR PC segmentation in RS. The authors summarize publicly available 3D datasets for deep learning training and testing and report performance values obtained when benchmarking the methods surveyed on widely used datasets. The authors state that the dynamic graph CNN (DGCNN) and ConvPoint outperform other CNN models in remote sensing applications while maintaining lightweight structures.

Three categories of PCSS techniques—projection-based, voxel-based, and direct point-based techniques—are presented in detail and compared by the authors of [15], who also provide a comprehensive overview of their development. Within the framework of PCSS, each approach has a distinct use case. It is argued that when high-performance computing systems are not available, projection-based techniques are the best option because they prioritize computational efficiency over performance. According to the authors, voxel-based techniques are appropriate for 3D object classification because they capture the entire context, while point-based methods work well for applications such as 3D SS and are excellent at capturing fine features.

#### 2.1.3. Three-Dimensional Mesh Processing

Recent DL architectures for 3D sensed data processing, including segmentation, object detection, and classification, are reviewed by the authors of [16]. Background ideas, conventional techniques, and contemporary methods and representation modalities such as meshes, RGB-D, multi-view, volumetric, ordered, and unordered point clouds are covered. The study also provides a descriptive list of datasets available for each type of representation. The report finishes with a thorough analysis of deep learning’s prospects for processing 3D sensed data, emphasizing the areas that would benefit most from more research.

The article by Adam et al. [17] offers a thorough summary of recent advancements in DL algorithms for SS in 3D meshes representing scenes at the urban scale. Several mesh-based learning methods are described, generalizing DL algorithms on mesh surfaces. Along with a discussion of benchmark large-scale mesh datasets and a comparative analysis of the evaluated approaches, evaluation tools for assessing segmentation performance are provided.

#### 2.1.4. Point Cloud Registration

The authors of [18] provide a thorough evaluation of feature-based coarse registration and fine registration techniques for LiDAR data in photogrammetry and RS. The methods considered are based on coarse features and include surface-based, line-based, and point-based techniques. The fine registration techniques described are iterative approximation techniques (i.e., iterative closest point), normal distribution transforms (NDTs), random sample consensus (RANSAC [28]), and techniques utilizing auxiliary data. As explained in [29], the RANSAC algorithm can be also used in combination with the scale-invariant feature transform (SIFT) to enhance registration efficiency. A comprehensive review of current advancements in RANSAC-based methods can be found in [30]. The absence of uniform assessment procedures and standard data has been noted as a major drawback as it impedes a fair comparison between methods.

A thorough overview of the concepts and techniques for DL-based mismatch reduction is provided by the authors of [19]. The authors provide an overview of several network designs, geometric information extraction methods, and training modes. The authors list current mining techniques, describe their permutation invariant features, and highlight the significance of permutation invariance in these operations. In order to clarify the principles and efficacy of widely used techniques, both intuitive and mathematical analyses are offered.

An extensive description of DL-based PC registration is detailed by the authors of [20]. This review provides insights from four different angles: the attention mechanism, graph convolutional network, multi-layer perceptron, and deep neural network. Also included is a comprehensive analysis of registration performance measures and datasets related to point cloud registration based on deep learning.

#### 2.1.5. Multispectral Remote Sensing Data

Recent fusion techniques for optical images and LiDAR utilized in photogrammetry and RS are reviewed by the authors of [21]. Many techniques, including real orthophotograph creation, pan-sharpening, key target recognition, registration, classification, change detection, 3D reconstruction, and forest inventory, are presented for data fusion in a variety of applications.

The authors of [22] assess the most recent techniques for unmanned aerial vehicles (UAVs) spectral RS, including geometric processing, sensor technologies, measurement protocols, and radiometric calibration. The authors explain the trajectory of reflected energy as it travels from particles to being represented as pixels in 3D spectral point clouds, surface maps, or 2D maps.

A comprehensive review of recent multispectral LiDAR technologies and their uses is provided by the authors of [23]. The applications covered include topographic mapping, change detection, ecology and forestry, bathymetry, objects and land use/land cover (LULC) categorization, geology and archaeology, and navigation.

### 2.2. Remote Sensing in Specific Point Cloud Applications

This subsection provides an overview of several review papers dedicated to specific RS-related PC applications. The articles surveyed are listed in Table 2, which adopts the same format as Table 1.

#### 2.2.1. Point Clouds in Urban Model Reconstruction and Building Information Modeling

The authors of [31] explore methods that may be used to reconstruct 3D models of urban objects from PCs, including vegetation; buildings; utilities such as electricity lines, roads, and bridges; and free-form architectural features such as statues and curved buildings.

The authors of [32] offer a thorough analysis of cutting-edge point-cloud-based urban scene reconstruction approaches, with a focus on data collection and the advantages and disadvantages of important processing techniques. The authors review various techniques for acquiring, organizing (points, voxels, and patches), registering, and reconstructing point clouds in three dimensions.

The authors of [33] describe the approach of scanning for building information modeling (BIM). Photogrammetry and LiDAR procedures for creating point clouds are covered in the paper. It also compares LiDAR systems mounted on diverse platforms, including airborne, spaceborne, mobile, and terrestrial ones, and discusses the advantages of combining data from several sources. Additionally, thorough explanations of several PC processing techniques—such as registration, sampling, SS, and compression—are given. In addition, compression methods such as SPR-PCC [53] and projection-based algorithms [54] are surveyed, as described later in Section 4.

#### 2.2.2. Road Detection and Extraction in Remote Sensing

An overview of mobile LiDAR technology is provided by the authors of [34], who cover geometrical accuracy validation, data error analysis, direct georeferencing, and system components. A review of studies on road information inventory is carried out with an emphasis on finding and extracting road surfaces, minor structures, and pole-like objects.

The authors of [35] provide a comprehensive review of road extraction techniques using 2D images and 3D LiDAR point clouds. The authors classify these methods into three main categories—2D, 3D, and fused approaches—with additional sub-grouping within each category.

#### 2.2.3. Power Line Modeling

The authors of [36] discuss the benefits and drawbacks of using cutting-edge LiDAR scanning equipment and examine the advantages and disadvantages of several techniques for 3D electrical power line corridor inspection. Their study focuses on techniques for extracting and reconstructing power lines by surveying research articles devoted to that problem, especially those published in conferences and journals related to geosciences. This survey shows that image and PC-based methods are becoming more popular for detecting, locating, segmenting, and inspecting power lines, enabling the automation of tasks related to routine power line inspection and maintenance operations.

#### 2.2.4. Urban Object Change Detection

The most recent advancements in PC data-based urban object change detection are reviewed by the authors of [37]. Thanks to developments in structure-from-motion (SfM) photogrammetry and LiDAR technologies, 3D change detection utilizing PC data has attracted substantial attention recently. The article offers a thorough examination of applications related to four categories of urban objects: construction sites, street scenes, structures, and urban trees. The evaluation pays more attention to open-source datasets that incorporate change labels and provides an overview of how various data sources are used for each type of object.

#### 2.2.5. Infrastructure Management and Structural Damage Mapping

The evolution of UAV-based structural damage mapping is reviewed by the authors of [38], which moves from basic descriptive overviews to complex texturing and segmentation algorithms and deep learning techniques. Machine learning, enhanced autonomy in mapping, damage mapping in GPS-denied environments, infrastructure maintenance, and robotic UAVs are some of the emerging innovations summarized in this article.

A comprehensive review of LiDAR technology for both commercial and research applications is given in survey [39], along with information on its uses in critical infrastructure monitoring. It covers the monitoring of distribution pipelines for water, oil, and gas; energy production facilities; and ground and air transportation. It also provides an overview of the LiDAR datasets that are currently available for these applications. PCL compression [55], described in Section 4, was proposed as an solution for the problem of PC transmission over Wi-Fi.

#### 2.2.6. Shoreline Mapping

A comprehensive assessment of the literature on shoreline mapping published from 2000 to 2021 is presented by the authors of [40], who attempt to find and analyze research topics and patterns pertaining to shoreline change detection. The authors come to the conclusion that, in light of the significance of safeguarding communities in delta, coastal, and riverine regions, it is imperative to address research gaps in shoreline change analysis by posing new questions and utilizing newer instruments and technologies such as artificial intelligence and machine learning. According to the authors, UAVs, PC data for shoreline change analysis, and high-resolution satellite imagery might all be used as techniques for achieving centimeter-level accuracy.

An overview of coastline mapping using aerial LiDAR is presented by the authors of [41], which covers the availability of data, laser scanning equipment, and current extraction approaches throughout the past 20 years. The authors conclude that there are still data availability issues and some limitations inherent in the technology when it comes to using aerial LiDAR for coastline mapping. Still, many opportunities exist for improvement, particularly when paired with LiDAR point cloud processing methods based on deep learning algorithms.

#### 2.2.7. Landslide Detection

The authors of [42] review typical remote-sensing techniques for landslide assessment, with an emphasis on their applicability to hazard detection and monitoring while taking location and survey costs into consideration. The overview discusses systems that are terrestrial, airborne, and spaceborne and outlines the advantages and disadvantages of each for the acquisition, analysis, and interpretation of data. The presented examples include lasergrammetry, terrestrial optical photogrammetry, and interferometric synthetic aperture RADAR (InSAR).

#### 2.2.8. Point Cloud Segmentation of Discontinuous Plane Surfaces

The benefits, drawbacks, and capabilities of different segmentation algorithms for surface extraction from discontinuity planes are reviewed by the authors of [43], who also discuss the difficulties specific to the processing of PC data representing rock faces. Analyses of segmentation and orientation results from studies on two rock mass surface PC datasets are presented, and some recommendations for generating consistent and repeatable ground truth orientations are given.

#### 2.2.9. Point Cloud Semantic Segmentation for Specific Remote-Sensing-Related Tasks

The use of small-footprint LiDAR sensors for high-resolution aerial RS applied to urban land cover SS is reviewed by the authors of [44]. The conclusion is that satellite RS has been shown to be effective for monitoring land cover on a wide scale; however, more research has to be carried out on finer-scale maps, particularly in metropolitan areas, as this has been demonstrated by a number of biophysical and socioeconomic studies. The article also covers the use of compression methods in the applications surveyed, namely, LASzip [56], LCMP [57], lossy LAS [58], and octree-based compression [59] (some of which are reviewed in Section 4).

The integration of PCSS with the workflow of historical building information modeling (HBIM) is reviewed by the authors of [45], in a survey study. The article summarizes a few dozen studies covering automatic and semi-automatic methods and tools for geometric modeling applied to HBIM.

#### 2.2.10. Space Exploration and Remote Sensing Applications

The authors of [46] examine the sensor choice and application of the Mars InSight Lander (Interior exploration utilizing Seismic Investigations, Geodesy, and Heat Transport). Image products are used extensively in many lander tasks, such as processing raw telemetry; making mosaics; creating terrain meshes; stereo correlation; radiometric correction; and producing various products such as instrument deployment maps, surface normals, PC data, and layers for roughness maps.

#### 2.2.11. Remote Sensing in Aquatic Environments

The authors of [47,48] cover twelve distinct RS systems that are frequently used in ocean research: four passive (optical systems, thermal infrared radiometers, microwave radiometers, and global navigation satellite system reflectometry) and eight active (SAR, scatterometers, altimeters, LiDAR, gravimeters, SONAR, high-frequency RADAR, and marine RADAR) systems. A thorough evaluation and discussion were conducted on 15 applications of RS in the ocean, utilizing various RS systems and approaches: ocean surface wind, ocean wave height, ocean surface current, ocean tide, ocean surface salinity, ocean color, ocean chlorophyll, ocean oil spills, underwater ocean, sea level, sea ice, icebergs, sea surface temperature, ship detection, and fisheries.

The authors of [49] provide an overview of airborne oceanic LiDAR RS technology and applications. Multi-channel airborne LiDAR devices are intended to greatly enhance the resolution and quality of data for marine biological and geographic profiles. In order to encourage further study in ocean biogeochemistry, algorithms for biological product retrieval and modeling based on common radiation transfer models are described.

The study presented in [50] marks a significant advancement in the autonomous and precise operation of deep-sea autonomous underwater vehicles (AUVs) near the seabed, focusing on enhancing underwater terrain-aided navigation (TAN) techniques. TAN leverages underwater terrain features as reference points for positioning. It enables the real-time localization of AUVs within pre-existing terrain maps by actively detecting and tracking distinct terrain characteristics, maintaining positioning errors constrained within temporal and spatial domains. The article explores the background, operational principles, and key technical aspects of underwater TAN. It reviews the algorithms central to the two primary modules of TAN: the terrain-aided positioning module and the iterative filtering estimation module.

#### 2.2.12. Virtual and Augmented Reality for Remote Sensing Applications

An application of virtual reality/augmented reality (VR/AR) applications in RS involves telepresence robots, which are increasingly recognized for their role in enhancing social interactions [51]. An example of remote collaborative systems called “BeHere”, where co-presence can be useful for instructions based on virtual replicas, combining gestures and avatars for procedural tasks, is explained by the authors of [52]. In this case, RGB-D frames are encoded and transmitted to the remote side, where they are decoded and reconstructed into PCs.

### 2.3. Agriculture-Related Applications

This subsection surveys and synthesizes the contents of several review studies focused on the use of PCs and related representations in remote sensing applications to agriculture. The studies are listed in Table 3 in the usual format.

#### 2.3.1. General Use

The authors of [60] discuss the use of unmanned aircraft systems (UASs) to affect remote imaging for improved farming operations such as field mapping, chemical spraying, biomass estimation, plant stress detection, weed control, and inventory counting. Different tools and technologies, such as PCs, vegetation indices, machine learning algorithms, and statistical methods, are crucial to precision agriculture.

The authors of [61] examine research on the use of LiDAR systems, such as MLSs, aerial laser scanners (ALSs) and terrestrial laser scanners (TLSs), in precision agriculture, with a focus on crop cultivation. Subsequently, they showcase current LiDAR uses, particularly in digitizing trees and plants, estimating crop-related metrics, planning and decision assistance, and object detection and navigation.

#### 2.3.2. Forestry

Several articles are specific to forestry applications of remote-sensing-based on PCs.

The methods for delineating individual tree crowns from 3D data and their applications in ecology and forestry are reviewed by the authors of [62]. It is determined that while approaches utilizing the entire point cloud are required to identify smaller trees beneath the canopy, 2D surface model methods (derived from point clouds) are often the best for detecting taller trees.

The current status of national forest inventory and forest management in the Nordic countries is examined by the authors of [63], who also highlight the advantages and disadvantages of different RS materials and data-gathering techniques from the viewpoints of various audiences.

The authors of [64] evaluate the technical prerequisites for generating high-quality measurements from autonomous platforms with various drone aircraft and commercial laser scanners. An example of an autonomous helicopter in a Southern Czech Republic’s temperate mountain forest is also included in the case study.

The possible application of consumer-grade cameras and unmanned aerial vehicles for terrestrial SfM-based surveys in forestry is covered by the authors of [65]. The authors show that with the help of the SfM workflow, foresters can gather several RS datasets, using a single sensor to generate multiple spatial products.

The authors of [66] summarize current knowledge about the ecological significance of the European aspen, talk about the challenges associated with understanding the species’s occurrence and dynamics in boreal forests, and look at the possibilities presented by different RS technologies for aspen mapping.

The authors of [67] summarize research on the categorization of tree species using data from aerial laser scanning, identifying the best classification algorithms and the most useful features generated from LiDAR. It is argued that the most accurate features are those derived from full-waveform data, while radiometric features mixed with height data also work well. Furthermore, according to the publications reviewed, the best results for species discrimination are obtained with support vector machines and random forest classifiers.

Alvites et al., in [68], look at the classification and quantification of timber assortments using terrestrial and aerial LiDAR devices, including UASs. When it comes to describing understory trees, terrestrial LiDAR systems perform quite well. For comprehensive timber assortment data over huge forest areas, combining terrestrial technologies with airborne/UAS LiDAR looks promising. Furthermore, there is increased interest in these approaches, as observed via the increasing usage of machine and DL algorithms in analyzing LiDAR data.

The authors of [69], Demol et al., compare data from ten TLS-derived above-ground biomass (AGB) investigations with values based on destructive tree harvesting. It was concluded that AGB obtained from TLS closely matches values from destructive analyses.

The authors of [70] examine feasible alternatives for developing targets for landscape forest restoration that take spatial patterns into account. The hierarchical levels of a forested surrounding are represented via spatial patterns in analyses. The division of the landscape is carried out hierarchically: sub-catchments, vegetation patches, and individual trees.

The authors of [71] describe distinct instances of DL methods in diverse forest applications and classify them based on their processing techniques and operational principles. Several sensors and equipment used to collect data on forests are introduced. The authors also list and provide details about forest imagery datasets that are currently accessible and investigate the global geographic distribution of the related research.

#### 2.3.3. Vegetation Parameter Extraction

Using satellite thermal images, the authors of [72] investigate the merits and limitations of the most widely used models for determining plant water stress and soil moisture. They also report a number of indicators, such as the normalized differential vegetation index, also applied to assess soil moisture, in addition to evapotranspiration.

Basic leaf area index (LAI) retrieval techniques, validation procedures, and constraints are reviewed by the authors of [73], employing point cloud data from aerial LiDAR scanners. The gap fraction model and empirical regression are the two primary LAI retrieval method types evaluated. The empirical and gap fraction models’ poor scalability over time, space, and various airborne LiDAR systems is demonstrated via empirical validation.

The authors of [74] investigate different facets of vegetation parameter extraction with TLS, such as retrieval techniques and parameters extracted from TLS point clouds. Primary and secondary vegetation parameters are examined. The primary parameters are computed directly from point clouds, whilst the secondary parameters are approximated from the primary ones.

The common applications of vegetation cover fraction (fCover) in a variety of fields, settings, and scales are presented by the authors of [75]. Along with traditional non-imaging techniques, the review includes LiDAR return-based techniques (e.g., return intensity retrieval, return number index), image-based techniques (e.g., spectrum retrieval, unmixing, segmentation), and PC-based techniques (e.g., rasterization) on different platforms.

#### 2.3.4. Viticulture

Precision viticulture potential and uses are covered by the authors of [76]. The explanation of various sensor types and their working principles covers both proximal and RS platforms, such as satellites and UAVs. The study includes descriptions of vegetation status indicators used in viticulture, as well as supervised and unsupervised techniques for image classification and segmentation. Additionally, it investigates photogrammetric techniques for dense PC-based 3D canopy modeling. The study also highlights how large-scale datasets may be processed and analyzed using deep learning and machine learning approaches to evaluate the physiological and agronomic biomarkers of vineyards.

#### 2.3.5. Weed Management

Dobbs et al. examine the applications of 3D imaging technologies in [77], such as photogrammetry, SfM, and LiDAR, in weed management. The authors explore the applications of 3D photogrammetric imaging in weed management, such as weed detection and mapping for targeted removal, and in weed-related research, such as modeling weed–crop competition, to predict yield loss. They also examine the use of 3D imaging for weed management in orchards and grasslands.

## 3. Point Cloud Datasets for Remote-Sensing-Related Tasks

**Figure 3:**
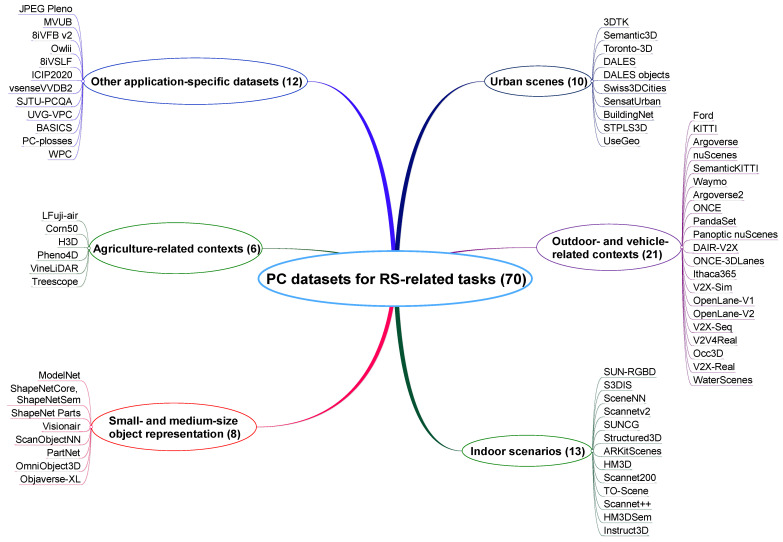
Map of discussed point cloud datasets for remote-sensing-related tasks.

This section provides an overview of various datasets that are particularly relevant to tasks related to RS. These datasets are diverse in nature and are categorized based on the type of PC data. To facilitate a more structured understanding, we classify the datasets into six distinct categories, as depicted in Figure 3:

- Urban scenes: This category includes datasets of urban environments, such as buildings, roads, and public infrastructure. These datasets are used for applications such as urban planning or traffic management.
- Outdoor- and vehicle-related contexts: Datasets in this category cover large-scale outdoor environments and vehicle-centric data, often used in autonomous driving research and outdoor navigation systems. They typically include data from roads, highways, and surrounding landscapes.
- Indoor scenarios: These datasets focus on enclosed environments such as homes and offices. They are typically used for applications in robotics and interior mapping.
- Small-size and medium-size object representation: This category is dedicated to datasets capturing smaller objects and simple shapes. It is used for object recognition, segmentation, and shape understanding.
- Agriculture-related contexts: Datasets here represent agricultural landscapes, including crops and fields. These are typically used in precision agriculture and crop monitoring.
- Other application-specific datasets: This category includes datasets that do not fit into the above categories. These datasets are commonly used in research studies on compression methods and visual quality evaluations.

Each of these datasets acts as a reference for the compression methods that will be discussed in further detail in Section 4. Categorization also helps determine which dataset is most suited for particular RS-related tasks, improving the effectiveness of compression methods designed for each type of PC data.

Table 4 summarizes 10 urban scenario datasets, referencing studies from 2017 to 2024, including one online repository, that are frequently referenced in research for evaluating and comparing different algorithms. Each dataset is accompanied by a brief description to highlight its key features and applications. It can be observed that most datasets are primarily designed for semantic segmentation. The UseGeo dataset [78] can be used for 3D reconstruction from images, supporting both single-image depth estimation and multi-view 3D reconstruction, with LiDAR data serving as a reference. Other tasks can be also performed, including image orientation, feature extraction and matching, the automated registration of images with LiDAR data, and semantic segmentation. The 3DTK repository [79] offers a diverse collection of point clouds, primarily intended for testing and developing PC registration algorithms. However, some datasets within the repository can also be utilized for change detection and object detection. Several PCs include additional attributes such as color, thermal, and reflectance information.

Table 5 summarizes 21 outdoor- and vehicle-related datasets, referencing papers from 2011 to 2024. A short description of each dataset is also given. Global navigation satellite system (GNSS) and inertial measurement unit (IMU) information is present in most datasets, except for the Waymo and ONCE datasets, which provide similar information: vehicle poses at different points in time (both) and translational and angular velocity (Waymo).

Table 6 summarizes 13 indoor-scene datasets, referencing papers from 2015 to 2023, that are frequently utilized in research articles for benchmarking and evaluating various algorithms. Each dataset is accompanied by a concise description, highlighting its primary characteristics and typical applications.

Table 7 summarizes eight datasets featuring 3D objects, referencing papers from 2015 to 2023, that are widely used in research articles to benchmark and compare different algorithms. Each dataset is accompanied by a brief description, summarizing its key features and typical research applications.

Table 8 summarizes six datasets related to agriculture that are frequently referenced in research articles, referencing papers from 2020 to 2024. Each dataset is briefly described, highlighting its core features and specific agricultural applications and providing a clear understanding of their suitability for various research tasks.

Table 9 summarizes 12 specific datasets, referencing studies from 2016 to 2023, including one online repository, that are frequently utilized in research studies focused on compression techniques and visual quality assessments. Each dataset is briefly described, showcasing its relevance and use cases in the context of PC data processing and evaluation.

## 4. Point Cloud Compression

This section provides an overview of some of the current PC compression models and methods, with the aim of providing additional information to fill the gaps left by most of the review papers covered in this survey. Two articles surveyed in this text, refs. [6,8], are exceptions in this aspect as they describe and compare some point cloud compression methods. An important aspect of PC compression methods is the evaluation of the distortion introduced by the (lossy) coding, usually through the use of objective measures that compare the quality of the reconstructed PC with that of the original PC. A good amount of information about these measures can be found in [149,150].

**Figure 4:**
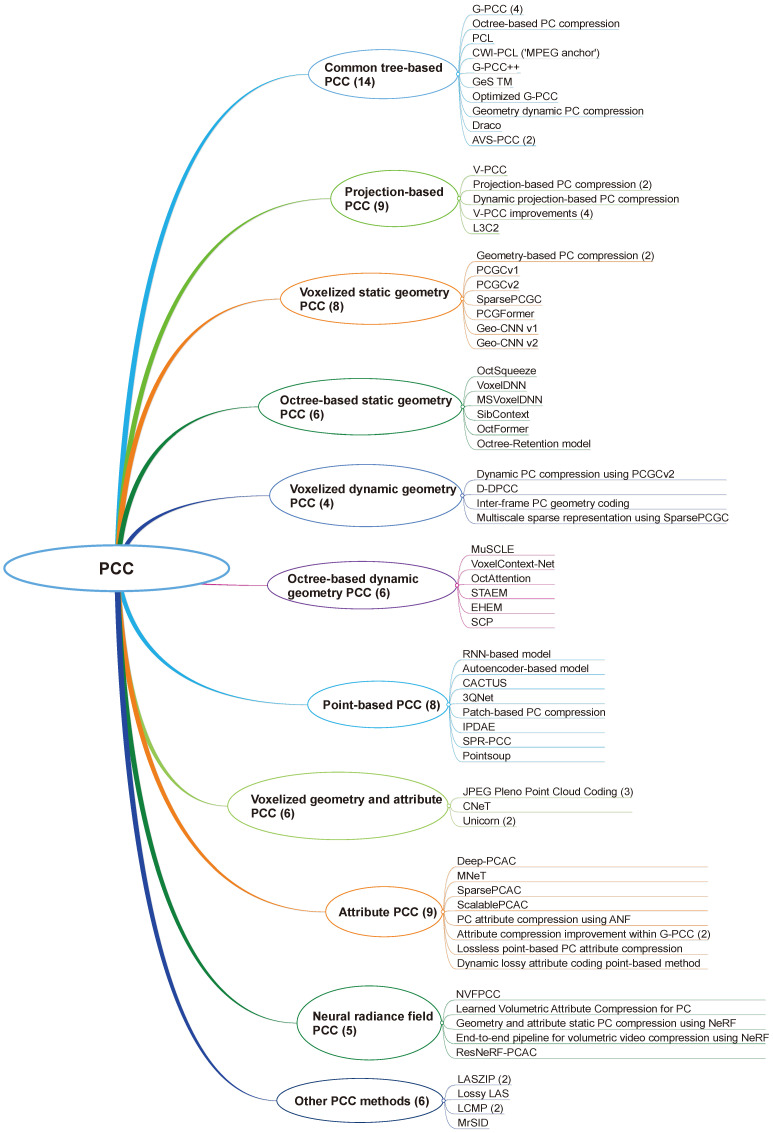
Map of discussed point cloud compression algorithms (PCC—point cloud compression).

We classify the compression methods into several categories with some common properties, as shown in Figure 4, in which the main categorization is related to the basic coding principle. While general lossless data compression algorithms such as ZIP or RAR can be applied to PCs, they are not specifically designed for this purpose and are therefore not included in this survey.

Table 10 provides an overview of the PC compression methods and their specific application types and use cases inferred mostly from the datasets employed in the reviewed studies. The methods listed are compared relative to several properties: the type of point cloud (static and dynamic), point cloud components (geometry and attributes), and compression fidelity (lossless and lossy).

The “Dynamics” column in Table 10 indicates whether the method is applied to static, dynamic, or both types of PCs, based on the tested cases in the reviewed studies. Methods labeled with only “static” could potentially be used in dynamic scenarios but do not exploit temporal redundancy between PCs. Conversely, only “dynamic” signifies that the model is explicitly designed for dynamic PCs, considering temporal redundancies. The “L/LS” (lossy/lossless) column in Table 10 indicates whether the method can be used for lossy or lossless compression fidelity or both. In some cases, both methods can be used.

The next subsections describe each of the mentioned compression methods in more detail, while the last subsection discusses PC compression applications.

### 4.1. Common Tree-Based Point Cloud Compression

To construct the compressed point cloud, G-PCC [26] encodes the content directly in 3D space. Geometry and attribute data are encoded independently in G-PCC. Since geometry is necessary for attribute coding, geometry coding is firstly applied. Coordinate transformation, voxelization, and either an octree or a trisoup (“triangle soup”) surface approximation are the first steps in the geometry encoding process. Finally, to attain lower bitrates, arithmetic coding is used. Three choices are offered for attribute coding: a lifting transform, a predicting transform, and the region-adaptive hierarchical transform (RAHT). Upon applying one of these transforms, the coefficients undergo quantization and arithmetical encoding. The authors of [141,201,202] contain further information regarding G-PCC.

Other prior studies based on octree can be also found, i.e., [59], for lossless compression. The point cloud library (PCL) [55] offers an octree-based method for compressing point clouds.

The CWI-PCL (“MPEG anchor”) codec is discussed in [152], based on the point cloud library (PCL) [55], for dynamic PC geometry and attribute compression, and it is suitable for 3D real-time tele-immersion. Newer methods based on G-PCC have also been recently proposed, such as G-PCC++ [203], which addresses compression distortion and improves quality compared to the original G-PCC. In another paper based on G-PCC [204], the authors propose a solution to optimize the octree codec of G-PCC to be more precise. GeS TM is also proposed [151] as a branch derived from the G-PCC test model for compressing static and dynamic solid PCs.

Another open-source library called Draco [143], based on kd-tree, can also be used to compress and decompress PCs, as well as 3D geometric meshes.

The authors of [205] propose an approach that investigates redundancies between the successive frames of a dynamic PC sequence in order to reduce geometry information in a lossless manner. It operates by gradually increasing the octree’s resolution. Experimental results show better performance than Draco and CWI-PCL (in a lossless mode), for example, using MVUB [136] and 8iVFB v2 [137] datasets.

AVS (Audio Video Coding Standard Workgroup of China) established a subgroup in 2019, AVS-PCC, to efficiently compress LiDAR point clouds that are both static and dynamic in different scenarios: autonomous driving, cultural heritage, and dynamic scenarios [153]. Recently, geometry and attribute bit rate allocations were optimized for precise bit control by the authors of [206]. More details about AVS-PCC are provided by the authors of [201].

### 4.2. Projection-Based Point Cloud Compression

Using V-PCC coding [156], the PC is first divided into multiple connected regions to generate 3D surface segments. Such 3D surface segments are called "patches" and are subsequently projected one at a time into a 2D patch. Reducing projection issues such as occlusions and hidden surfaces is made easier using this technique. Each 2D patch consists of a set of images: a binary image, a geometry image (depth map), and attributes of the projected points. H.265/HEVC video compression is employed to compress the 2D sequences with the packed patches after they are generated, although any other compression method may also be utilized. More details about V-PCC are provided by the authors of [141,201].

Projection-based techniques were applied previously, as demonstrated by the authors of [154], who presented a projection-based PC compression algorithm. The authors of [54] introduce a projection-based PC compression strategy comprising four techniques: range image conversion, PC segmentation, prediction, and coding. These methods were evaluated using the KITTI dataset [24]. The authors of [155] developed a new dynamic PC compression method that combines surface reconstruction with various projection types and bit depths with latter video compression to produce geometry and texture maps. Recently, some papers also proposed improvements for V-PCC. The authors of [207] propose voxel selection-based refining segmentation to accelerate the PC-refining segmentation process, as well as data-adaptive patch packing to reduce occupancy map size. The authors of [208] propose an efficient geometry surface coding method to improve geometry information compression in V-PCC. The authors of [209] propose a lightweight, fully connected network-based fast CU size decision during H.265/HEVC utilization for V-PCC. The authors in [210] propose a method for segmenting dynamic point clouds based on shape similarity and occlusion before generating patches. The experimental results indicate that the proposed method outperforms V-PCC and some other existing methods for both geometric and texture data.

A low-latency, low-complexity codec (L3C2) was recently proposed by MPEG [27]. L3C2 was developed for the rotating LiDAR sensor, storing points such as a coarse and then residual 2D projection in polar coordinate systems. It can be used for both lossy and lossless coding, as well as for geometry and attribute compression.

### 4.3. Voxelized Static Geometry Point Cloud Compression

The authors of [157] present geometry-based compression called DSAE (Distributed Source AutoEncoder), which divides input data into 8×8×8 voxel blocks. These blocks are then represented by the encoder using a deep syndrome (which corresponds to the autoencoder’s hidden variables). The decoder then reconstructs the coded PC using coded features (deep syndrome data) and side information from the side information encoder. An improved version of PC geometry compression is presented by the authors of [158] using adversarial distributed source autoencoders.

Another study [161] comprises an improved version of PCGCv1 and PCGCv2 [159,160], called SparsePCGC. In SparsePCGC, the convolutions are only carried out on sparsely distributed, most probably positively occupied voxels. The proposed model uses a sparse convolution-based neural network (SparseCNN) and occupancy probability approximation model based on SparseCNN in order to calculate the occupancy probability in a single-stage or in a multi-stage manner. SparsePCGC can be used for both lossless and lossy geometry compression. The proposed approach demonstrates excellent performance across a variety of datasets, including sparse LiDAR PC geometry (SemanticKITTI [91] for training and testing and the Ford dataset [88] for testing) and dense object PC geometry (JPEG Pleno dataset [135,137], Owlii dataset [138], and MVUB dataset [136]; Shapenet dataset [122] for training), compared to G-PCC and other DL-based models while having low complexity. The same authors propose lossy PC geometry compression in [162] using transformer networks stacked with sparse convolutions, showing better results than G-PCC.

The authors of [163] provide a lossy PC geometry compression technique called Geo-CNN for static point clouds that uses uniform quantization and learned convolutional transforms. An improved version, Geo-CNN v2, is also presented [164].

### 4.4. Octree-Based Static Geometry Point Cloud Compression

The authors of [165] propose OctSqueeze, which initially encodes LiDAR points into an octree, an effective data structure appropriate for PCs with sparse points. The probabilities of the octree symbols are then modeled in a conditional entropy model with a tree structure, which encodes the octree into a compact bitstream. The experimental results are compared with Draco [143] and CWI-PCL codec [152] using two datasets, the newly created NorthAmerica (proposed by the same authors) and SemanticKITTI [91] datasets, showing better results for the proposed codec.

The authors of [166] demonstrate a context-adaptive arithmetic coding-based DL lossless compression technique for static PC geometry, called VoxelDNN. The proposed encoder functions in a hybrid mode that combines voxel-based and octree coding. The experimental results on ModelNet [121], MVUB [136], and 8iVFB v2 [137] show better results compared to G-PCC v12. A multiscale approach, MSVoxelDNN, is presented in [167], speeding up encoding and decoding times compared to VoxelDNN.

An innovative multi-level framework based on octrees is presented in [168] for large-scale sparse and unstructured PC compression. By utilizing the context of neighbors, ancestors, and siblings’ children, the framework employs a novel entropy model to explore hierarchical dependency in an octree. Experimental results using the SemanticKITTI [91] and nuScenes [90] datasets show better results compared to G-PCC, Draco, and VoxelContext-Net.

The authors of [169] propose OctFormer, an octree-based transformer compression technique that does not rely on sibling nodes’ occupancy data. The proposed approach builds octree node sequences using non-overlapping context windows and distributes the outcome of a multihead self-attention operation over multiple nodes. Experiments using the SemanticKITTI [91] and ScanNet [111] datasets show the better performance of the OctFormer model compared to G-PCC and OctSqueeze. VoxelContext-Net (without coordinate refinement models, i.e., postprocessing) exhibits a similar performance to OctFormer on the ScanNet dataset and somewhat lower performances with respect to SemanticKITTI for higher bitrates, and performance enhancements that are several times higher are also observed. OctAttention exhibits similar reconstruction quality on the SemanticKITTI dataset; however, OctFormer’s decoding time is much faster.

The authors of [170] present the Octree-Retention model. Initially, the point cloud objects are segmented using an octree structure. Then, important features are extracted from sibling and ancestor nodes using octree-based contextual windows. Finally, prior knowledge between spatially nearby nodes can be successfully used for compression using the Octree-Retention model, which uses retentive networks (RetNet). Experiments show better performance compared to a) G-PCC, VoxelContext-Net, OctAttention, and OctFormer for the SemanticKITTI [91] dataset (lossy mode) and b) G-PCC, VoxelDNN, and OctAttention for the 8iVFB v2 [137] dataset (lossless mode).

### 4.5. Voxelized Dynamic Geometry Point Cloud Compression

The authors of [171] present dynamic PC geometry compression using variational autoencoders with temporal autoregressive hyperprior and sparse convolutions, with the PCGCv2 model [160] used for individual PCs. Improved geometrical quality is realized compared to the V-PCC described earlier. Using 3D motion estimation, inter-frame geometry PC coding, and compensation in the feature space, the authors of [172] suggest a unique 3D sparse convolution-based deep dynamic point cloud compression (D-DPCC) network to compress and adjust the dynamic PC geometry, also showing better performance than V-PCC inter-frame coding. This is tested on the 8iVFB v2 human body dataset [137]. The authors of [174] use a multiscale sparse representation (MSR) framework from static PCs to compress dynamic PC geometry, advancing the static SparsePCGC encoder [161]. The suggested approach achieves lower bpp values in comparison to G-PCC and SparseGCPC in a lossless mode, and it realizes BD-rate gains in the lossy mode compared to V-PCC, SparsePCGC, PCGCv2, D-DPCC, and the methods reported by the authors of [173].

### 4.6. Octree-Based Dynamic Geometry Point Cloud Compression

To save storage space for LiDAR sensor data streams, the authors of [175] describe a compression algorithm, MuSCLE, which takes advantage of spatiotemporal relationships across many LiDAR sweeps. A new conditional entropy model is suggested to represent the likelihood of the octree symbols by considering coarse-level geometry and geometric and intensity information from previous sweeps. Afterwards, the complete data stream is encoded into a compact one. The experimental results using the SemanticKITTI [91] and NorthAmerica (proposed by the same authors) datasets show better performances compared to the OctSqueeze, Draco, CWI-PCL, and G-PCC codecs.

The authors of [176] suggest VoxelContext-Net, a two-stage deep learning system, for both dynamic and static point cloud compression. The suggested method combines the advantages of voxel-based schemes and octree-based techniques by compressing octree structured data using the voxel’s context. The experimental results using two datasets, SemanticKITTI [91] (static and dynamic case) and ScanNet [111] (static case), compared with OctSqueeze, G-PCC, and Draco, show better results for the proposed codec.

The authors of [177] present OctAttention, a multi-context deep learning codec that makes use of the memory-efficient octree structure for point clouds. Through the collection of sibling and ancestor nodes, the proposed method encodes octree symbol sequences. Experiments that use static LiDAR PC via SemanticKITTI [91] show the better performance of the proposed model in the lossy mode compared to VoxelContext-Net (without coordinate refinement models, i.e., postprocessing) and Octsqueeze. Moreover, using dynamic MVUB [136] and 8iVFB v2 [137] results in better outcomes in the lossless dynamic case compared to VoxelDNN, MSVoxelDNN, G-PCC v1, and the model reported by the authors of [205].

The authors of [178] suggest a learning-based entropy model, STAEM (Spatiotemporal Attention Entropy Model), for dynamic PC compression that takes advantage of the large-scale spatiotemporal context based on octrees. The authors provide a graph-based feature extraction methodology that takes geometry into account in order to extract useful features from a large-scale, informative context. Moreover, a spatiotemporal attention mechanism is presented by the authors to identify dependencies in the large-scale context. The experimental results with respect to SemanticKITTI [91] (static and dynamic cases) show better results, compared to OctAttention, OctSqueeze, and G-PCC in the static case and G-PCC and MuSCLE in the dynamic case. Moreover, using dynamic MVUB [136] and 8iVFB v2 [137] results in better outcomes in the lossless dynamic case compared to VoxelDNN, G-PCC, and OctAttention.

To improve the efficiency of the octree-based auto-regressive entropy model, the authors of [179] suggest an EHEM (efficient hierarchical entropy model), a hierarchical attention structure that preserves the global receptive field and exhibits linear complexity relative to the context scale. In addition, the authors provide a grouped context structure that maintains compression efficiencies while resolving the auto-regression-related serial decoding problem. The experimental results using the SemanticKITTI [91] and Ford [88] datasets show better performances in the dynamic case compared to the OctAttention, SparsePCGC, and G-PCC models while exhibiting a decoding latency that is similar to the effective conventional G-PCC model.

The authors of [211] provide spherical coordinate-based learning PC compression (SCP), a model-agnostic technique that takes advantage of the point clouds’ many azimuthal angle invariance features and circular shapes. Furthermore, in order to reduce the reconstruction error for far-off regions inside the spherically coordinate-based octree, the authors suggest a multi-level octree for SCP. The experimental results that use the SemanticKITTI [91] and Ford [88] datasets show better performances when using SCP with EHEM and OctAttention compared to the baselines, SparsePCGC, and G-PCC.

### 4.7. Attribute Point Cloud Compression

Several studies have dealt with PC static attribute compression. The authors of [189] present end-to-end deep lossy point-based PC attribute compression, called Deep-PCAC, assuming that the geometry is coded with some existing geometry codecs. A multiscale lossless (or lossy) voxelized PC attribute coding method called MNeT is presented in [190], assuming lossless geometry coding.

The authors of [212] present an approach called SparsePCAC that uses sparse convolutions to create a variational autoencoder (VAE) framework for compressing PC attributes, assuming lossless geometry coding. The experimental results show that SparsePCAC performs better than G-PCC v6 and existing DL methods. The authors of [191] propose a scalable PC attribute compression method called ScalablePCAC, assuming lossless geometry coding. It uses G-PCC as the base layer and a model as an enhancement layer, showing better performances than G-PCC v14, v22, and SparsePCAC.

The authors of [192] provide a PC attribute compression strategy based on the augmented normalizing flow (ANF) model, including sparse convolutions and assuming lossless geometry coding. In comparison to VAE-based coding schemes, the normalizing flow model’s invertibility allows for improved reconstruction. The experimental results show better performances compared to G-PCC v14, Deep-PCAC, and SparsePCAC.

The authors of [213] present an embedded attribute PC encoding method based on SPIHT as an alternative to the RAHT transform within G-PCC. Furthermore, the authors of [214] propose a scalable, embedded PC attribute encoding based on a multilayer perceptron used with an RAHT transform within G-PCC.

The authors of [193] present lossless point-based PC attribute compression, assuming lossless geometry compression, and this was tested using a diverse set of PCs: objects (i.e., ShapeNet [122]), indoor scenes (i.e., ScanNet [111]), JPEGs (i.e., 8iVFB v2 [137]), and LiDAR (SemanticKITTI dataset [91]). Better performance was observed compared to G-PCC, MNeT [190], and CNeT [186].

An end-to-end learned dynamic lossy attribute coding point-based method is presented in [194], assuming lossless geometry compression, which uses effective high-dimensional convolution to capture complex inter-point correlations. The experimental results show better results compared to region-adaptive hierarchical transform (RAHT) attribute compression models within the G-PCC codec.

### 4.8. Voxelized Geometry and Attribute Point Cloud Compression

The JPEG Pleno Point Cloud Coding codec was recently introduced in [25], and it is currently used as Verification Model V4.0 [215,216], which has joint geometry and an attribute-coding system. Additionally, the optional DL module performs upsampling/super-resolution to enhance the final quality of decompressed PCs. Details about the training and test PCs for the comparison between submitted models can be found in [149]. The training dataset consists of the ShapeNet dataset [122] and different PCs, both static and dynamic, from JPEG or MPEG providers, while in the test dataset, there are 12 defined PCs with three types of PCs present: solid, dense, and sparse. These represent different density classes. Lossless static PC geometry and attribute compression models (CNeT and Context NeTwork) were presented in [186] and tested on human-body datasets (i.e., 8iVFB v2 [137], Owlii [138]).

Unicorn [187,188] is a newer learning-based solution designed to compress static and dynamic, geometrical, and attribute PCs with different source characteristics (such as 8iVFB v2 [137], Owlii [138], JPEG Pleno [135], KITTI [24], Ford [88], and Scannet [111] datasets) in both lossy and lossless modes. It realizes significantly better performance than standard methods such as MPEG G-PCC, V-PCC, and other learning-based approaches, delivering state-of-the-art compression efficiency with a practical level of complexity.

### 4.9. Point-Based Point Cloud Compression

Several studies have been conducted that use direct PCs as inputs. A method utilizing a recurrent neural network and residual blocks to gradually compress the data from a single frame of 3D LiDAR is presented by the authors of [180].

Other papers have proposed autoencoders for generative purposes; however, they may also be used for compression-related tasks. For example, the authors of [181] use autoencoder models to learn the compact representation of a PC, while generative adversarial networks (GANs) (with both raw PC data and latent space data) and Gaussian mixture models (GMMs) (with latent space data) are studied to generate novel PCs.

The authors of [182] propose a coding system called Content-Aware Compression and Transport Using Semantics (CACTUS), which divides the original PC into independent streams using SS with the RandLA-Net [217] architecture. The segments are then encoded with DSAE, G-PC, or Draco compression.

A novel DL-based PC compression model is proposed in [183], called the 3D PC Geometry Quantization Compression Network (3QNet), which can handle dense points; it can overcome the existing point-based approaches’ robustness issue. Experiments related to Visionair [124], ScanNet [111], and SemanticKITTI [91] show that 3QNet is capable of achieving better compression efficiencies than CWI-PCL, G-PCC, Draco, PCGCv2, and Geo-CNN.

The authors of [184] propose an IPDAE model (improved patch-based deep autoencoder), which incorporates several improvements over the patch-based point cloud compression method described by the authors of [218] (inspired from PointNet [219]). These consist of octree coding for centroid point sampling, a learnable context model for entropy coding, and an integrated training and compression procedure. The experimental results using ModelNet [121], ShapeNet [122], and S3DIS [109] show better outcomes compared to G-PCC, PCGCv2, Geo-CNN, and Geo-CNN v2.

The authors of [53] propose scene-aware LiDAR PC geometry compression using semantically prior representation (SPR-PCC) by projecting PCs to 2D images, segmenting projection images, and eventually removing moving objects from a set of projected frames (depending on the final application task). The proposed solution shows better results compared to G-PCC v14 and PCL using the KITTI dataset [24].

The authors of [185] present Pointsoup, an effective learning-based geometry codec that simultaneously realizes both very low decoding latencies and high performances. A point model-based approach that uses dilated window-based entropy modeling and an attention-based encoder inspired by the traditional Trisoup codec was introduced. The experimental results using S3DIS [109], ScanNet [111], and SemanticKITTI [91]—compared to G-PCC v23 (octree and trisoup), OctAttention, IPDAE, and 3QNet—showed better results for the proposed codec.

### 4.10. Neural Radiance Field Point Cloud Compression

The novel-view synthesis approach NeRF (neural radiance field) [220], which is also applicable to PC compression, is covered in this subsection. NeRF was first proposed to reconstruct a 3D scene from sparse 2D representations, i.e., to synthesize novel views of a scene. NeRF approaches can be classified into three types based on how they depict the scenes: implicit, explicit, and hybrid [221]. Explicit and hybrid radiance field representations alleviate the slowness of implicit representations by including explicit data structures (such as 2D/3D grids or 3D points) for local feature encoding. The same study introduces binary radiance fields (BiRFs), a storage-efficient hybrid model of radiance fields that uses binary feature encoding. Another storage-efficient NeRF method was recently proposed by the authors of [222], called context-based NeRF compression (CNC), following the design of state-of-the-art binary radiance field (BiRF) compression. The experimental results on the Synthetic-NeRF (synthetic) [220] and Tanks and Temples (real) [223] datasets showed the better performance of the proposed CNC method compared to the BiRF.

PC geometry compression using NeRF was presented by the authors of [195], called NVFPCC, for both static and dynamic PCs. The authors of [196] present learned volumetric attribute compression (LVAC) for PCs using coordinate-based networks. The authors of [197] present a unified approach for geometry and attribute static PC compression using NeRF. Two networks were used for the geometry and attribute components. The voxelized body dataset from the authors of [137] were used to carry out comparisons with existing solutions, namely, the G-PCC standard and NVFPCC, showing better performances for the proposed solution. PCs from the Semantic3D dataset [80] were also compared to those reported by the authors of [160,162], showing better performances when using geometry quality assessments. The authors of [198] propose an end-to-end pipeline for volumetric video compression utilizing neural-based representation. Three-dimensional dynamic content is represented as a sequence of NeRFs, which are converted from explicit to neural representations. The experimental results show better performances than G-PCC and Draco, and similar performances are observed relative to the V-PCC coding solutions using the 8iVFB v2 [137] and 8iVSLF [139] datasets. The authors of [199] present a new method for point cloud attribute compression called residual neural radiance fields for point cloud attribute coding (ResNeRF-PCAC). Tests on the 8iVFB v2 [137] dataset show better results than G-PCC; the region-adaptive hierarchical transform (RAHT); and pred/lift schemes for attribute coding, v14 and v21.

### 4.11. Other Point Cloud Compression Methods

In this subsection, we explore several additional compression methods that are not addressed in the previously discussed subsections.

The lossless compression scheme for LiDAR in the binary LAS (LASer) data format, LAZ (LASzip), is presented by the authors of [56,224], achieving 7–20% of the uncompressed size. The LASzip compressor is lossless, non-progressive, streaming, and order-preserving, and it provides random access. Another lossless LiDAR PC compression method, the LAS compression coder (LCMP), was presented by the authors of [57,225], with only 10–20% of their original size. Lossy LAS file compression using uniform space division was presented by the authors of [58]. A commercially available compression format multi-resolution seamless image database (MrSID) is also available for images and LAS LiDAR files for both lossless and lossy compression [200].

### 4.12. Point Cloud Compression Applications

In this subsection, we discuss different PC compression applications, mostly based on the employed datasets, as described in Table 10. The most common PC applications comprise virtual reality/augmented reality (VR/AR) with MVUB [136], 8iVFB v2 [137], and Owlii [138] datasets, as well as autonomous driving with the KITTI [24] and SemanticKITTI [91] datasets. For VR/AR applications, trained datasets are usually sampled objects from the ModelNet [121] and ShapeNet [122] datasets, which means that those models should also work for these datasets. In the case of the ANF-based model for attribute compression [192] and Pointsoup for geometry compression [185], the dataset used in model training is mentioned because its type is completely different from the test datasets; this is carried out to test their generalization abilities in compressing different PC types.

Less prevalent tested datasets include indoor scene datasets (usually ScanNet [111]) and urban scenario datasets (i.e., Semantic3D [80], 3DTK [79]), used only in a few proposed compression algorithms. Regarding the datasets with object PCs, the sampled ModelNet [121] and ShapeNet [122] datasets are usually used to train DL-based models.

In non-DL based models, standardized codecs such as G-PCC [26], V-PCC [156], and AVS-PCC [153] are versatile, supporting both static and dynamic PCs with lossless and lossy options and several application types. Specialized compression tools such as LASzip [56] and MrSID [200] focus on LiDAR PC compression. Draco [143] can be used in telepresence and VR/AR due to efficient geometry and attribute encoding. The newly proposed L3C2 codec can be used in autonomous driving.

From Table 10, it can also be observed that DL-based models combining geometry and attribute compression are less common compared to geometry-only models. Notable examples include lossless CNeT [186], lossy JPEG Pleno PCC [25], and lossy models [197] for static PCs, as well as lossy models [198] for dynamic PCs.

It is also notable that some DL-based methods either omit the exact G-PCC version used for comparison or rely on older versions available at the time of evaluation. This can be attributed to the rapid development of the G-PCC and V-PCC codecs, with the latest versions possibly not being publicly accessible. Additionally, comparisons often focus only on similar codec types, overlooking other DL-based methods. Since different codecs are tailored to specific PC types, as indicated by the datasets they use (e.g., dense PCs such as 8iVFB v2 [137] or sparse PCs such as SemanticKITTI [91]), it is essential to consider and specify the exact PC type when comparing methods against the G-PCC codec.

A summary of the advantages and disadvantages of each PC compression group described earlier is provided in Table 11.

### 4.13. Point Cloud Compression Limitations and Research Trends

As summarized in Table 11, the PC compression methods reviewed have some drawbacks. In general, the compression efficiency in lossy modes is still not very high, especially when high fidelity (i.e., low geometrical distortion) is needed, as in the case of remote sensing applications involving some metrology operations. At low and medium coding rates (i.e., low bits per point), well-established methods based on geometry processing such as octree-decomposition can introduce point position errors that are incompatible with applications in which accurate measurements are to be carried out from decoded/reconstructed point clouds. To address this problem, research is ongoing with respect to the compression of point clouds in order to attain near-lossless performance where the distortion allowed is measured via the min–max criteria (i.e., ensuring bounded maximum positional errors computed over all point cloud points) instead of the currently used average point-to-point or point-to-surface errors. Deep-learning-based solutions, despite showing promising performance and, in some cases, surpassing that of alternative solutions, also exhibit specific problems, such as the introduction of points representing artificial structures during reconstruction/decompression, possibly impacting the operation of downstream processing. Learning-based solutions also suffer from complexity ails, usually requiring fast and energy-expensive parallel processing hardware for acceptable coding and decoding time-complexity, somewhat limiting the contexts in which they can be deployed. Concerning scalability and random access functionalities, which are necessary, for instance, to decompress and render the representations of compressed 3D point clouds at different scales and/or in different parts of the encoded point clouds without decoding the entire point cloud, in general, all methods developed thus far only support these functionalities in a limited fashion. Most methods, both deep-learning-based and non-deep-learning-based, resort to block partitioning and encoding in order to provide some sort of random access, allowing the independent decoding of each block. In the case of scalability, some compression methods such as MPEG G-PCC and V-PCC support some scalability, but most deep-learning-based methods have no provisions for scalable coding. A notable exception is the method described by the authors of [187], which can support scalable encoding and decoding. Thus far, most research on point cloud compression has focused on the efficient representation of geometrical information, which is, without a doubt, the most important component for most remote sensing applications. However, attribute information that can represent surface temperatures, surface reflectances, etc., is also very important and quite hard to represent efficiently. Recognizing this, several research groups (e.g., those reported in [188]) and standard organizations such as ISO/IEC JPEG and MPEG are working on this side of the point cloud compression problem. It is expected that the results of these research efforts will translate into more compact compressed 3D point clouds that are easier to store and process, with advantages for remote sensing applications and processing workflows.

## 5. Conclusions and Future Research

This article is organized into three main sections: a meta-review of review papers on the RS application of point clouds (Section 2), datasets commonly used in RS-related algorithm research and development (Section 3), and PC compression methods (Section 4).

Section 2 surveys a selection of review articles about the applications of PC technology in RS-related contexts, with articles divided into three groups: general PC-related, specific RS-related, and agriculture-related applications. The first group of articles cover PC acquisition and processing tasks, such as scene understanding, compression, segmentation, registration, multispectral applications, and multimodal data fusion. Some of these processing tasks are not exclusive to remote sensing and are also used in, e.g., computer vision applications relative to several problems. The second and third groups of this article are more specifically related to RS tasks, with the third group further divided for agriculture-related applications. Table 1, Table 2 and Table 3 summarize each discussed article. The range of applications covered in the section, as well as the number of studies selected for review, shows that point clouds are used in many application scenarios that fall under the umbrella of remote sensing.

Section 3 provides a list of datasets (point clouds and related) used for the research of PC compression methods. Section 4, as well as research in other areas, refers to the following topics: autonomous vehicle navigation (outdoor- and vehicle-related contexts), robotics and interior mapping (indoor scenarios), precision agriculture (agriculture-related contexts), and other application-specific datasets for visual quality evaluations and related objective quality measures. Many of the mentioned datasets are also used in PC object detection, as well as semantic, instance, and panoptic segmentation. Datasets containing 2D RGB (+depth) images are also suitable for image segmentation tasks and for the fusion of 2D and 3D data. More detailed information regarding each dataset can be found in Table 4, Table 5, Table 6, Table 7, Table 8 and Table 9 and the articles referenced in those tables. Overall, this section shows that there is now a reasonably large set of 3D point cloud and related dataset formats, and in many cases, they are accompanied by application-specific annotations, which are very useful for anyone wanting to carry out research on remote sensing problems.

Section 4 provides information regarding different PC compression methodologies that researchers have developed; these methodologies aim to provide efficient point cloud data transmission, handling, and storage that are usable in remote sensing applications. The methods surveyed are divided into several categories: common tree-based, projection-based, and other methods. The methods are able to process voxelized or unprocessed real coordinate point clouds in both static or dynamic scenarios. Besides PC geometry component compression, some methods also address attribute compression. Emerging representation formats such as neural radiance fields and Gaussian splattings are also covered. Several standardized point cloud coding methodologies, such as MPEG’s geometry-based (G-PCC) and video-based (V-PCC) codecs, and recent DL-based models, such as the JPEG Pleno Point Cloud Coding codec, are included in this survey.

As point cloud technology evolves, it is expected that new datasets, algorithms, and hardware improvements will further enhance its application in remote sensing technologies and systems. Future research should concentrate on creating even more effective compression techniques that balance data volume and representation fidelity, in addition to investigating how deep learning models may help optimize point cloud data processing. Compression algorithms for both geometry and attribute data (potentially utilizing radiance-field synthesis) could be explored, considering different application contexts such as urban scenarios, indoor scenes, autonomous driving on water surfaces (i.e., the WaterScenes dataset [107]), and agriculture-related environments. Both lossy and lossless compression methods can also be explored. New algorithms could be tested using both real and synthetic datasets created via generative tools, such as Objaverse-XL [128], with respect to both real and synthetic objects. Due to their flexibility and economy of representation, point clouds will continue to be essential for improving remote sensing capabilities and applications as they are used to tackle the issues identified in this survey.

## Figures and Tables

**Table 1:** Summary of recently published review papers describing general PC processing for RS tasks: MS/HS—multispectral/hyperspectral; SS—semantic segmentation; RGB-D—depth generated from stereo/multiview structure-from-motion (SfM) or depth cameras.

Application	Short Description	Scanner Type	Platform Type	Paper (Y)
General analysis and processing of RS data	General and RS PC processing tasks: scene understanding, compression, and completion	RGB-D, LiDAR	Ground, aerial	[6] (2022)
	General analysis of 2D/3D RS data	RGB-D, LiDAR, SAR, MS/HS	Aerial, satellite	[7] (2022)
	General and RS PC processing tasks: acquisition, processing, and engineering applications	RGB-D, LiDAR, MS/HS	Ground, aerial, spaceborne	[2] (2023)
	Compression methods for automotive LiDAR PC	LiDAR	Ground	[8] (2024)
	Compression methods for automotive LiDAR PC with analysis of impact on object detection	LiDAR	Ground	[9] (2024)
RS applications in scene understanding	PC processing, general, and in RS: feature extraction, object detection, and SS	RGB-D, LiDAR	Ground	[10] (2019)
	PC segmentation, general, and in RS	RGB-D, LiDAR, SAR, MS/HS	Ground, aerial	[11] (2020)
	SS of images and PC	RGB-D, LiDAR, SAR, MS/HS	Aerial, spaceborne	[12] (2021)
	PC segmentation, general, and in RS	LiDAR	Aerial	[13] (2021)
	PC segmentation, general, and in RS	LiDAR	Ground, aerial	[14] (2022)
	PC processing, general, and in RS: classification, detection, and segmentation	RGB-D, LiDAR	Ground, aerial	[15] (2024)
3D mesh processing	PC and mesh processing, general, and in RS: classification, detection, and segmentation	RGB-D, LiDAR	Ground, aerial	[16] (2019)
	SS of 3D meshes	RGB-D, LiDAR	Ground, aerial	[17] (2023)
PC registration	Registration of LiDAR data	LiDAR, MS/HS	Ground, aerial	[18] (2018)
	Image/PC matching in computer vision and RS tasks	Not specified	Not specified	[19] (2023)
	PC registration, general, and in RS	RGB-D, LiDAR	Ground, aerial	[20] (2024)
Multispectral RS data	Fusion of RGB-D and LiDAR data in different RS applications	RGB-D, LiDAR, MS/HS	Ground, aerial, spaceborne	[21] (2017)
	Spectral RS measurements	RGB-D, LiDAR, MS/HS	Aerial	[22] (2018)
	Multispectral LiDAR applications in RS	LiDAR, MS/HS	Ground, aerial, spaceborne	[23] (2024)

**Table 2:** Summary of recently published review papers describing specific RS-related PC applications: MS/HS—multispectral/hyperspectral; BIM—building information modeling; SS—semantic segmentation; RGB-D—depth is generated from stereo/multiview structure-from-motion (SfM) or depth cameras; VR/AR—virtual reality/augmented reality.

Application	Short Description	Main Scanner Type	Platform Type	Paper (Y)
PC to urban model reconstruction and BIM	3D urban model reconstruction from PC	RGB-D, LiDAR	Ground, aerial	[31] (2018)
	3D urban model reconstruction from PC	RGB-D, LiDAR, SAR, MS/HS	Ground, aerial	[32] (2021)
	Building information modeling	RGB-D, LiDAR	Ground, aerial, spaceborne	[33] (2022)
Road detection and extraction in RS	Road information inventory	RGB, LiDAR, MS/HS	Ground, aerial	[34] (2016)
	Road extraction in RS	RGB-D, LiDAR, SAR, MS/HS	Ground, aerial, spaceborne	[35] (2022)
Power line modeling	Power line modeling	RGB-D, LiDAR	Ground, aerial	[36] (2023)
Urban object change detection	Urban object change detection	RGB-D, LiDAR	Ground, aerial	[37] (2023)
Structural damage mapping	Structural damage mapping	RGB-D, LiDAR	Aerial	[38] (2019)
	Critical infrastructure monitoring using LiDAR	LiDAR, MS/HS	Ground, aerial	[39] (2023)
Shoreline mapping	Shoreline mapping	–	Aerial, spaceborne	[40] (2022)
	Shoreline mapping	LiDAR	Aerial	[41] (2023)
Landslide detection	Landslide detection	RGB-D, LiDAR, SAR, MS/HS	Ground, aerial, spaceborne	[42] (2020)
PC segmentation of discontinuous plane surfaces	PC segmentation of discontinuous plane surfaces	RGB-D, LiDAR	Ground, aerial	[43] (2022)
PC SS for specific RS-related tasks	Urban land cover SS	RGB, LiDAR, MS/HS	Aerial, spaceborne	[44] (2015)
	PC SS in heritage building information modelling	RGB-D, LiDAR	Ground, aerial	[45] (2023)
RS and other data analysis in space	RS and other data analysis in space and Mars InSight lander	RGB-D	Space	[46] (2019)
RS and other data analysis in water	RS systems frequently used in ocean research	RGB, LiDAR, SAR, SONAR, MS/HS	Ground, aerial, spaceborne, shipborne	[47] (2022)
	RS systems frequently used in ocean research	RGB, LiDAR, SAR, SONAR, MS/HS	Ground, aerial, spaceborne, shipborne	[48] (2023)
	Airborne oceanic LiDAR RS	LiDAR	Aerial	[49] (2023)
	Autonomous terrain-aided navigation of deep-sea underwater vehicles	RGB-D, LiDAR, SONAR	Underwater	[50] (2024)
VR/AR applications in RS	Telepresence robots	RGB-D, LiDAR	Ground	[51] (2022)
	“BeHere”: collaboration system based on virtual replicas	RGB-D	Ground	[52] (2023)

**Table 3:** Summary of recently published review papers describing agriculture-related PC processing in RS: MS/HS—multispectral/hyperspectral; RGB-D—depth generated from stereo/multiview structure-from-motion (SfM) or depth cameras.

Application	Short Description	Main Scanner Type	Platform Type	Paper (Y)
General agriculture	Agriculture	RGB-D, LiDAR, MS/HS	Aerial	[60] (2019)
	Agriculture	LiDAR	Ground, aerial, satellite	[61] (2023)
Forestry	Delineation of individual tree crowns	RGB-D, LiDAR, MS/HS	Aerial	[62] (2017)
	Forest management in Nordic countries	RGB-D, LiDAR, MS/HS	Aerial, satellite	[63] (2018)
	Forest RS using drone and LiDAR	LiDAR	Aerial	[64] (2019)
	SfM photogrammetry for RS data in forestry	RGB-D	Aerial	[65] (2019)
	RS analysis of European aspen in boreal forests	RGB-D, LiDAR, MS/HS	Ground, aerial, satellite	[66] (2020)
	Tree species’ classification	LiDAR, MS/HS	Aerial	[67] (2021)
	Timber assortments	LiDAR	Ground, aerial	[68] (2022)
	LiDAR versus destructive harvesting to quantify above-ground biomass	LiDAR	Ground	[69] (2022)
	Forest restoration in RS	RGB-D, LiDAR, MS/HS	Ground, aerial, satellite	[70] (2022)
	Forest resource assessment	RGB-D, LiDAR, MS/HS	Ground, aerial, satellite	[71] (2024)
Vegetation parameter extraction	Soil moisture and plant water stress	Thermal image	Satellite	[72] (2016)
	Leaf area index (LAI) retrieval	LiDAR	Aerial	[73] (2021)
	Extraction of vegetation parameters in savanna biome	LiDAR, MS/HS	Ground	[74] (2021)
	Vegetation cover fraction (fCover)	RGB-D, LiDAR, MS/HS	Ground, aerial, satellite	[75] (2023)
Viticulture	Precision viticulture	RGB-D, LiDAR, MS/HS	Ground, aerial, satellite	[76] (2023)
Weed management	Weed management in RS	RGB-D, LiDAR, MS/HS	Ground, aerial	[77] (2022)

**Table 4:** Summary of recently published studies describing urban-level datasets: N. CL—number of classes; N. P—number of points; RSM—real/synthetic/mixed; TLS—terrestrial laser scanner; MLS—mobile laser scanner; ALS—aerial laser scanner; UAV-P—UAV photogrammetry; UAV-L—UAV LiDAR; SS—semantic segmentation; IS—instance segmentation; DE—depth estimation.

Dataset Name	Platform	N. CL	N. P	Area ($m^{2}$) or Length (m) Covered	Average Point Density (pts/$m^{2}$)	RSM	Task Suitability	Short Description
3DTK [79]	Several	–	–	–	–	R	PC registration	Repository for 3D point clouds from robotic experiments
Semantic3D [80]	TLS	8	$4\;\times \;10^{9}$	–	Varying	R	SS	Wide range of urban outdoor scenes
Toronto-3D [81]	MLS	8	$78.3\;\times \;10^{6}$	$1\;\times \;10^{3}$ m	1000	R	SS	PC dataset of Toronto, Canada
DALES [82]	ALS	8	$505\;\times \;10^{6}$	$10\;\times \;10^{6}$ $\;m^{2}$	50	R	SS	Dayton-annotated LiDAR earth scan (DALES)
DALES objects [83]	ALS	8	$492\;\times \;10^{6}$	$10\;\times \;10^{6}\;m^{2}$	50	R	SS, IS	DALES dataset [82] with additional intensity and IS
Swiss3DCities [84]	UAV-P	5.2	$3147\;\times \;10^{6}$ hi-res; $226\;\times \;10^{6}$ mid-res	$2.7\;\times \;10^{6}\;m^{2}$	1166 hi-res; 84 mid-res	R	SS	PCs from three cities in Switzerland: Zurich, Zug, and Davos
SensatUrban [85]	UAV-P	13 (31)	$2847\;\times \;10^{6}$	$7.64\;\times \;10^{6}\;m^{2}$	373	R	SS	PCs from three cities in UK: Birmingham with SS, Cambridge with SS, and York without SS
BuildingNet [86]	mesh, PC	31	$200\;\times \;10^{6}$	–	–	S	SS, classification	Labeled mesh and PC building parts; 2000 objects ($100\;\times \;10^{3}$ points per mesh) with $292\;\times \;10^{3}$ annotated components
STPLS3D [87]	UAV-P	9/9/9/9 real; 6/17/20 synthetic	–	$1.27\;\times \;10^{6}\;m^{2}$ real; $16\;\times \;10^{6}\;m^{2}$ synthetic	100 real; 11 synthetic	M	SS, IS	SS on four real datasets (nine different classes each); synthetic 3D data generation method with three IS datasets (6/17/20 different classes each)
UseGeo [78]	UAV-L, UAV-P	–	$392.8\;\times \;10^{6}$	$0.715\;\times \;10^{6}\;m^{2}$ (image area)	51	R	Multiview DE, monocular DE, feature extraction and matching, SS	UAV-based multi-sensor (RGB, LiDAR) datasets for geospatial research

**Table 5:** Summary of recently published papers describing outdoor- and vehicle-related datasets: RSM—real/synthetic/mixed; bbox—bounding boxes; Det—detection; Tra—tracking; MF—motion forecasting; SS—semantic segmentation; PS—panoptic segmentation; IS—instance segmentation.

Dataset Name	Type	RSM	Short Description
Ford [88]	LiDAR, $360^{\circ }$ RGB	R	3 sequences with 1500 scans each; on average, $100\;\times \;10^{3}$ points per scan.
KITTI [24]	LiDAR, RGB	R	Det 2D/3D: 7481/7518 train/test images/PC, 80,256 bbox for 3 (3) classes; Tra: 917 tracked objects for 2 classes.
Argoverse [89]	LiDAR, $360^{\circ }$ RGB, stereo	R	Det 3D: $22\;\times \;10^{3}$ scenes, $993\;\times \;10^{3}$ bounding boxes, 15 (17) classes; Tra: 113 scenes, each 15–30 s, 11,052 tracks, 15 (17) classes; MF: 324,557 scenes, each 5 s, $11.7\;\times \;10^{6}$ unique tracks, 1 class; Stereo: 6624 stereo pairs with ground truth depth.
nuScenes [90]	LiDAR, $360^{\circ }$ RGB RADAR	R	Det 2D/IS 2D: 93,000 images, $800\;\times \;10^{3}$ bbox and IS masks (foreground objects), 23 classes; Det 3D: $40\;\times \;10^{3}$ PC, $1.4\;\times \;10^{6}$ bbox, 10 (23) classes.
SemanticKITTI [91]	LiDAR	R	Det 3D: $23\;\times \;10^{3}$/$20\;\times \;10^{3}$ train/test PC; $682\;\times \;10^{3}$ bbox for 8 classes; SS 3D: $23\;\times \;10^{3}$/$20\;\times \;10^{3}$ train/test PC; $4,549\;\times \;10^{6}$ points for 25 (28) classes.
Waymo [92]	LiDAR, $360^{\circ }$ RGB	R	Det 2D/Tra: $1\;\times \;10^{6}$ images, $9.9\;\times \;10^{6}$ bbox, $256\;\times \;10^{3}$ unique IDs, for 3 classes; Det 3D/Tra: $230\;\times \;10^{3}$ PC, $12\;\times \;10^{6}$ bbox, $113\;\times \;10^{3}$ unique IDs, for 4 classes; Added: MF (103,354 scenes, each 20 s, $10.8\;\times \;10^{6}$, 3 classes), 2D video ($100\;\times \;10^{3}$ images) SS for 28 and PS for 3 classes, 3D SS for 23 classes.
Argoverse2 [93]	LiDAR, $360^{\circ }$ RGB, stereo	R	Det 3D/Tra: 1000 scenes, each 15s with 30 classes; “LiDAR” (unannotated): 20,000 scenes, each 30 s, with LiDAR, HD maps, pose; MF: 250,000 scenes, each 11 s, 10 classes, $13.9\;\times \;10^{6}$ unique tracks; Map change: 1000 scenes, each 45 s, with LiDAR, HD maps; 200 with map changes.
ONCE [94]	LiDAR, $360^{\circ }$ RGB	R	Det 2D: images from annotated PC, $769\;\times \;10^{3}$ bbox, 5 classes, unannotated $7\;\times \;10^{6}$ images; Det 3D: annotated 16,000 PC, $417\;\times \;10^{3}$ bbox, 5 classes, unannotated $1\;\times \;10^{6}$ PC.
PandaSet [95]	LiDAR, $360^{\circ }$ RGB	R	Det 3D: 8240 annotated PC from 103 scenes, 28 classes; SS 3D: 6080 annotated PC from 76 scenes, 37 classes.
Panoptic nuScenes [96]	LiDAR	R	SS 3D/PS 3D/Panoptic tracking: $40\;\times \;10^{3}$ PC, $1,1\;\times \;10^{9}$ points for 16 (32) classes.
DAIR-V2X [97]	LiDAR, RGB	R	Sensory inputs from vehicles, infrastructure, and collaborative vehicles–infrastructure: Det 2D/3D: 71,254 images/PC from vehicles and infrastructure, 10 classes, $1.2\;\times \;10^{6}$ bbox; SS 2D/3D: 71,254 images/PC from vehicles and infrastructure, 10 classes.
ONCE-3DLanes [98]	LiDAR, $360^{\circ }$ RGB	R	Annotated lanes in 2D and 3D from ONCE dataset [94].
Ithaca365 [99]	LiDAR, RGB	R	Repeatedly recorded: diverse scenes, weather, time, and traffic conditions: 2D: bbox, amodal IS, and road segmentation, 7000 images, 6 classes; Det 3D: 175 PC, 6 classes
V2X-Sim [100]	LiDAR, $360^{\circ }$ RGB-D	S	Simulated multi-agent perception dataset for collaborative autonomous driving, up to 5 vehicles and 1 set of infrastructure; SS 2D: 6 RGB cameras with $60\;\times \;10^{3}$ images, bird’s eye view; Det 3D/Tra: $10\;\times \;10^{3}$ PC with $26.6\;\times \;10^{3}$ bbox.
OpenLane-V1 [101]	LiDAR, $360^{\circ }$ RGB	R	Annotated lanes in 2D and 3D from Waymo dataset [92].
OpenLane-V2 [102]	LiDAR, $360^{\circ }$ RGB	R	Annotated lanes in 2D and 3D from Argoverse2 [93] and nuScenes [90] datasets.
V2X-Seq [103]	LiDAR, RGB	R	Det 3D/Tra: 15,000 images/PC from vehicle and infrastructure, 10 classes, 110 tracked objects per scene, 95 scenes; MF: 210,000 scenes, each 10s, 8 classes, with traffic light.
V2V4Real [104]	LiDAR, $360^{\circ }$ RGB	R	Real multi-agent perception dataset for collaborative autonomous driving, 2 vehicles with collaboration; Det 3D: $40\;\times \;10^{3}$ images, $20\;\times \;10^{3}$ PC with $240\;\times \;10^{3}$ bbox, 5 classes.
Occ3D [105]	LiDAR	R	3D voxel occupancy dataset semi-automatically labeled from Waymo [92] and nuScenes [90] datasets.
V2X-Real [106]	LiDAR, $360^{\circ }$ RGB	R	Real multi-agent perception dataset for collaborative autonomous driving, 2 vehicles and 2 sets of infrastructure with 4 collaboration combinations; Det 3D: $171\;\times \;10^{3}$ images, $33\;\times \;10^{3}$ PC with $1.2\;\times \;10^{6}$ bbox, 10 classes.
WaterScenes [107]	4D RADAR, RGB	R	2D: bounding box, pixel annotations, 7 classes, 54,120 objects; 3D: point-level RADAR PC annotations, 7 classes, 54,120 objects; Tasks: object detection, waterline segmentation, free-space segmentation, object tracking, SS, IS, PS, panoptic perception.

**Table 6:** Summary of recently published papers describing indoor-scene datasets: N. CL—number of classes; N. SC—number of scenes; RSM—real/synthetic/mixed; PS—panoptic segmentation; IS—instance segmentation.

Dataset Name	Type	N. CL	N. SC	RSM	Short Description
SUN-RGBD [108]	RGB-D, PC	800	10,355	R	Semantic annotation of 10,355 RGB-D scene images in 47 scenes
					categories with about 800 object categories; annotated 146,617 2D polygons and 64,595 3D bounding boxes
S3DIS [109]	PC	12	5	R	Stanford 3D indoor scene; semantic annotation of five indoor-area PCs with $215\;\times \;10^{6}$ points
SceneNN [110]	mesh, RGB-D	40	100	R	100 RGB-D video scenes, reconstructed, annotated with per-vertex and per-pixel labels, bounding boxes for 1482 objects, object poses
Scannetv2 [111]	mesh, RGB-D	20	1613	R	1513 RGB-D video scenes with 3D camera poses, reconstructed, 36,213 objects (18 classes) with bounding boxes; voxel segmentation (18/20 classes for IS/PS, respectively)
SUNCG [112]	mesh	84	49,884	S	49,884 valid floors, with contain 404,058 rooms and 5,697,217 object instances from 2644 unique object meshes covering 84 categories; used for the semantic scene completion
Structured3D [113]	mesh, RGB	–	3500	S	3D “primitive + relationship” structure annotations of 21,835 rooms in 3500 scenes; 196,000 photo-realistic 2D renderings of the rooms
ARKitScenes [114]	PC, RGB-D	–	5047	R	RGB-D and PC acquisition of 5047 indoor scans; PC with annotated object bounding boxes from 17 furniture categories
HM3D [115]	mesh; RGB	–	1000	R	Habitat–Matterport 3D: 1000 building-scale textured 3D mesh reconstructions (no segmentation)
Scannet200 [116]	PC	200	1513	R	3D IS based on ScanNet with 200 classes
TO-Scene [117]	mesh	52	16,077	M	16,077 scenes with real tables and 60,174 synthetic objects on; vertex segmentation, 3D bounding boxes, and camera poses
Scannet++ [118]	PC, RGB-D	>1000	460	R	460 3D reconstructions of indoor scenes with dense semantic and instance annotations; DSLR images and RGB-D sequences
HM3DSem [119]	mesh; RGB	1625	216	R	Habitat–Matterport 3D Semantic: 142,646 object instance annotations of 216 3D spaces with 3100 rooms
Instruct3D [120]	PC	–	280	R	280 scenes from Scannet++ [118] with approximately 10 different segmentation instructions, with 2565 instruction–point cloud pairings

**Table 7:** Summary of recently published papers describing object datasets: N. CL—number of classes; N. O.—number of objects; RSM—real/synthetic/mixed; IS—instance segmentation.

Dataset Name	Type	N. CL	N. O.	RSM	Short Description
ModelNet [121]	mesh	660	151,128	S	Annotated per model class
ShapeNetCore [122]	mesh	55	51,300	S	Annotated per model class
ShapeNetSem [122]	mesh	270	12,000	S	Annotated per model class; additional information is present
ShapeNet Parts [123]	mesh	16	31,963	S	Annotated parts, 42 labels for 16 classes, from ShapeNetCore dataset
Visionair [124]	mesh	–	60	S	Sixty distinct models, from rigid items (i.e., Chair) to smooth non-rigid objects (i.e., Bunny), downloaded from the Visionair repository
ScanObjectNN [125]	PC	15	15,000	R	From 700 scenes from SceneNN and Scannet selected 2902 unique objects; IS of 15 categories (with part annotations)
PartNet [126]	mesh	24	26,671	S	573,585 part instances with fine-grained, instance-level, and hierarchical 3D part information
OmniObject3D [127]	mesh; video	190	6000	R	Input meshes are rendered to PC and RGB-D images are included; COLMAP camera poses; rich text description of each object
Objaverse-XL [128]	mesh	–	>$10\;\times \;10^{6}$	M	10.2 million 3D deduplicated objects, coming from several sources, including metadata information, i.e., textual description

**Table 8:** Summary of recently published studies describing agriculture-related datasets (N. CL—number of classes; N. P/F—number of points/faces; ULS—UAV laser scanner; MLS—mobile laser scanner; SS—semantic segmentation).

Dataset Name	Platform	Type	N. CL	N. P/F	Short Description
LFuji-air [129]	MLS	PC	–	–	11 LiDAR-based PCs of Fuji apples trees with 1353 apple annotations
Corn50 [130]	–	PC	–	–	50 RGB PCs of artificial corn plants
H3D [131]	ULS	PC, mesh, RGB	11	73,909,354 P 8,550,338 F	LiDAR PCs and meshes of the village of Hessigheim, captured at four different epochs
Pheno4D [132]	ULS	PC	3	$260\;\times \;10^{6}$	PCs from 7 maize and 7 tomato plants over several days; Segmentation of “soil”, “stem”, and instance “leaf” points
VineLiDAR [133]	ULS	PC	–	356,633,530 P	10 3D LiDAR PCs in LASzip [56] format with RGB color
Treescope [134]	ULS, MLS	PC	–	–	SS and diameter estimation in agricultural environments: pine, oak, maple, and cedar forests; almond and pistachio orchards

**Table 9:** Summary of recently published papers describing specific datasets (N. O—number of objects; RSM—real/synthetic/mixed).

Dataset Name	Type	N. O	RSM	Short Description
JPEG Pleno [135]	PC, mesh	–	R	Diverse set of static and dynamic PCs for different tasks, such as static and dynamic PC compression
MVUB [136]	PC	10	R	JPEG Pleno Database: Microsoft Voxelized Upper Bodies (MVUB)— A Voxelized Point Cloud Dataset (dynamic)
8iVFB v2 [137]	PC	4	R	JPEG Pleno Database: 8i Voxelized Full Bodies (8iVFB v2)— A Dynamic Voxelized Point Cloud Dataset with 10-bit depth
Owlii [138]	PC	4	R	Owlii Dynamic Human Textured Mesh Sequence Dataset, 4 dynamic PCs
8iVSLF [139]	PC	6	R	8i Voxelized Surface Light Field (8iVSLF) Dataset— A Dynamic Voxelized Point Cloud Dataset with 12-bit depth
ICIP2020 [140]	PC	6	R	Static 6 original from [135] and 90 processed PCs, using two compression algorithms (G-PCC, V-PCC [141]) and octree pruning, for objective quality assessment
vsenseVVDB2 [142]	PC, mesh	8	R	Dynamic 4 PCs and 4 mesh sequences, compressed with Draco (for meshes) [143], G-PCC and V-PCC (for PCs) codecs [141] 152 distorted; for objective quality assessment
SJTU-PCQA [144]	PC	10	R	Static 10 original from [135] and 420 processed PCs, using 7 distortion types, for objective quality assessment
UVG-VPC [145]	PC	12	R	Dynamic voxelized PCs for visual volumetric video-based coding
BASICS [146]	PC	75	R	Static 75 original and more than 1200 processed PCs using 4 compression algorithms, for objective quality assessment
PC-plosses [147]	PC	4	R	Dynamic 3 original (from [135,138] ) and 105 processed PCs, V-PCC-compressed [141] and degraded by packet losses, for objective quality assessment
WPC [148]	PC	20	R	Static 20 original and 740 processed PCs, using 5 distortion types, for objective quality assessment

**Table 10:** Summary of PC compression methods and their applications: PCC—point cloud compression; S—static; D—dynamic; G—geometry; A—attribute; L/LS—lossy/lossless; VR/AR—virtual reality/augmented reality.

PCC Group	Model	Dynamics	PC Type	L/LS	PC Application (best for)
Common	G-PCC [26]	S,D	G,A	L,LS	Autonomous driving, cultural heritage
tree-based	GeS TM [151]	S,D	G,A	L,LS	VR/AR (solid PCs)
	Octree-based [59]	S	G,A	LS	Urban scenario
	PCL [55]	S	G,A	L,LS	Not specific
	CWI-PCL [152]	S,D	G,A	L	VR/AR, telepresence, “MPEG-anchor” codec
	AVS-PCC [153]	S,D	G,A	L,LS	Autonomous driving, cultural heritage, VR/AR
	Draco [143]	S	G,A	L,LS	VR/AR, telepresence, PC and mech compression
Projection-based	Projection-based [154]	S	G,A	L	Urban scenario
	Projection-based [54]	S	G	L,LS	Autonomous driving
	Projection-based [155]	D	G,A	L	VR/AR
	V-PCC [156]	S,D	G,A	L,LS	VR/AR, telepresence
	L3C2 [27]	S	G,A	L,LS	Autonomous driving
Voxelized	DSAE [157]	S	G	L	VR/AR
static	ADAE [158]	S	G	L	VR/AR, buildings
geometry	PCGCv1 [159]	S	G	L	VR/AR
	PCGCv2 [160]	S	G	L	VR/AR
	SparsePCGC [161]	S	G	L,LS	Autonomous driving, VR/AR
	PCGformer [162]	S	G	L	VR/AR
	Geo-CNN v1 [163]	S	G	L	VR/AR
	Geo-CNN v2 [164]	S	G	L	VR/AR
Octree-based	OctSqueeze [165]	S	G	L	Autonomous driving
static	VoxelDNN [166]	S	G	LS	VR/AR
geometry	MSVoxelDNN [167]	S	G	LS	VR/AR
	SibContext [168]	S	G	L	Autonomous driving
	OctFormer [169]	S	G	L	Autonomous driving, indoor scenes
	Octree-Retention [170]	S	G	L	Autonomous driving, VR/AR
Voxelized	Dynamic PCGCv2 [171]	D	G	L	VR/AR
dynamic	D-DPCC [172]	D	G	L	VR/AR
geometry	Dynamic PCC [173]	D	G	L	VR/AR
	Dynamic SparsePCGC [174]	D	G	L,LS	VR/AR
Octree-based	MuSCLE [175]	D	G	L	Autonomous driving
dynamic	VoxelContext-Net [176]	S,D	G	L	Autonomous driving, indoor scenes
geometry	OctAttention [177]	S,D	G	L,LS	Autonomous driving, VR/AR
	STAEM [178]	S,D	G	L,LS	Autonomous driving, VR/AR
	EHEM [179]	D	G	L,LS	Autonomous driving
Point-based	RNN-based [180]	S	G	L	Autonomous driving, urban scenario
	AE-based [181]	S	G	L	Simple objects
	CACTUS [182]	S	G	L	Autonomous driving
	3QNet [183]	S	G	L	Autonomous driving, indoor scenes, objects
	IPDAE [184]	S	G	L	Objects, indoor scenes, autonomous driving
	SPR-PCC [53]	S	G	L	Autonomous driving
	Pointsoup [185]	S	G	L	Indoor scenes, autonomous driving (trained only on objects)
Voxelized geometry	JPEG Pleno PCC [25]	S	G,A	L	VR/AR, buildings, cultural heritage, urban scenario
and attribute	CNeT [186]	S	G,A	LS	VR/AR
	Unicorn [187,188]	S,D	G,A	L,LS	VR/AR, autonomous driving, indoor scenes, buildings
Attribute	Deep-PCAC [189]	S	A	L	VR/AR, cultural heritage, buildings, indoor scenes
	MNeT [190]	S	A	L,LS	VR/AR
	ScalablePCAC [191]	S	A	L	VR/AR
	ANF-based [192]	S	A	L	VR/AR (trained only on indoor scenes)
	Model [193]	S	A	LS	Objects, indoor scenes, VR/AR, autonomous driving
	Model [194]	D	A	L	VR/AR
Neural	NVFPCC [195]	S,D	G	L	VR/AR
radiance	LVAC [196]	S	A	L	VR/AR
field	Model [197]	S	G,A	L	VR/AR, urban scenario
	Model [198]	D	G,A	L	VR/AR
	ResNeRF-PCAC [199]	S	A	L	VR/AR
Other PCC	LASzip [56]	S	G,A	LS	LiDAR LAS PC compression
methods	MrSID [200]	S	G,A	L,LS	LiDAR LAS PC compression

**Table 11:** Summary of advantages and disadvantages of reviewed PC compression methods (PCC—point cloud compression).

PCC Group	Advantages	Disadvantages
Common tree-based PCC	General category of many older and newer tree-based PCC models G-PCC represents newer standardized model [26] with rapid development	Depends on specific model
Projection-based PCC	General category of many older and newer projection-based PCC models V-PCC represents newer standardized model [156] with rapid development	Depends on specific model
Voxelized static geometry PCC	Better suited for dense PCs, i.e., in VR/AR applications	Works only for geometry PCs Temporal redundancies are not taken into account Input PC needs to be voxelized
Octree-based static geometry PCC	Better suited for large-scale sparse PCs, i.e, in autonomous driving applications	Works only for geometry PCs Temporal redundancies are not taken into account Decoding complexity
Voxelized dynamic geometry PCC	Better suited for dense PCs, i.e., in VR/AR applications	Works only for geometry PCs Input PC needs to be voxelized
Octree-based dynamic geometry PCC	Better suited for large-scale sparse PCs, i.e., in autonomous driving applications	Works only for geometry PCs Decoding complexity
Attribute PCC	In some cases, can be used instead or on top of existing solutions such as G-PCC	Usually, lossless geometry compression is assumed
Voxelized geometry and attribute PCC	Newer solutions usually based on deep learning models, as alternative to standardized codecs such as G-PCC and V-PCC	Training data have to be carefully chosen (i.e., depending on bit depth and motion for dynamic PCs)
Point-based PCC	Input PC does not have to be voxelized Generally works better for unevenly distributed and sparse point clouds	Complexity depends on number of points
NeRF PCC	Any volumetric input data can be used Images rendered with NeRF do not have visual artifacts due to PCs’ discrete nature Can be used to compress plenoptic PCs	Slower training time Fewer plenoptic PC datasets
Other PCC methods	Specific use cases for LiDAR PCC with specific type of PCC algorithm	Slower loading performance using compressed LAZ compared to uncompressed LAS, but also depends on software used [226]

## Data Availability

No new data were created in this manuscript.

## Abbreviations

The following abbreviations are used in this manuscript:

PC	Point cloud
PCC	Point cloud compression
RS	Remote sensing
LiDAR	Light detection and ranging
RADAR	Radio detection and ranging
SAR	Synthetic aperture RADAR
SONAR	Sound detection and ranging
DL	Deep learning
NeRF	Neural radiance field
SS	Semantic segmentation
SfM	Structure from motion
RGB-D	RGB plus depth
MLS	Mobile laser scanner
PCSS	Point cloud semantic segmentation
UAV	Unmanned aerial vehicle
UAS	Unmanned aircraft system
ALS	Aerial laser scanner
TLS	Terrestrial laser scanner
LAI	Leaf area index
fCover	Vegetation cover fraction
CNN	Convolutional neural network
IS	Instance segmentation
PS	Panoptic segmentation
VR/AR	Virtual reality/augmented reality
